# Metabolic reprogramming and M2 macrophage depletion define the microenvironment of adenomyosis

**DOI:** 10.3389/fendo.2025.1602814

**Published:** 2025-11-20

**Authors:** Xuejiao Bian, Zhe Sun, Junliang Lai, Boyu Li, Xinyi Dong, Hengyu Guan, Hugo Vankelecom, Yun Sun

**Affiliations:** 1 Department of Reproductive Medicine, Shanghai Key Laboratory for Assisted Reproduction and Reproductive Genetics, Ren Ji Hospital, Shanghai Jiao Tong University School of Medicine, Shanghai, China; 2 Laboratory of Tissue Plasticity in Health and Disease, Cluster of Stem Cell and Developmental Biology, Department of Development and Regeneration, Katholieke Universiteit (KU) Leuven, Leuven, Belgium

**Keywords:** adenomyosis, endometrium, immune infiltration, macrophage, metabolic flux, Keratan sulfate

## Abstract

**Background:**

Adenomyosis is a chronic gynecological disorder characterized by the invasion of endometrial tissues into the myometrium of the uterus, with pathophysiology linked to chronic inflammation and metabolic dysregulation. However, the understanding of the molecular mechanisms and underlying pathologies remains limited. This study aims to elucidate the metabolic reprogramming and immune dysregulation within the eutopic endometrium from patients diagnosed with adenomyosis, and identify potential therapeutic targets or diagnostic indicators.

**Methods:**

We analyzed publicly available microarray (GSE78851), bulk RNA-seq (GSE193928) and single-cell RNA-seq (Human Endometrial Cell Atlas) datasets to explore the microenvironment of eutopic endometrium from adenomyosis patients. Transcriptomic differences were assessed in GSE78851 (3 adenomyosis *versus* 5 controls), followed by immune composition and metabolic flux analysis. Findings on immune infiltration and metabolic changes were further validated in GSE193928 (6 adenomyosis *versus* 15 controls after quality control). Metabolic flux analysis was further extended to single-cell RNA sequencing data derived from endometrial samples of 63 donors. Key genes involved in keratan sulfate biosynthesis were further validated by RT-qPCR and immunofluorescence staining on endometrial biopsy samples.

**Results:**

Our study revealed widespread reprogramming in eutopic adenomyosis endometrium, characterized by enhanced immune-related pathways, reduction of M2 macrophage abundance, and disrupted metabolic processes. Further investigation of scRNA-seq data highlighted the cell type-specific metabolic profiles and immune-metabolic interplay within the endometrial microenvironment. Notably, the dysfunction of keratan sulfate biosynthesis, coupled with reduced M2 macrophage level, emerged as a consistent feature. Importantly, four key genes involved in keratan sulfate biosynthesis, including *CHST1*, *CHST6*, *B4GALT1*, and *B3GNT2*, were upregulated in eutopic endometrium compared to controls, suggesting their potential role in the pathophysiology of the disease.

**Conclusions:**

This study identifies dysregulation of keratan sulfate biosynthesis as a central feature of adenomyosis and links it to reduced M2 macrophage abundance and immune-metabolic imbalance. Validation of four keratan sulfate related genes strengthens their potential as biomarkers or therapeutic targets, providing novel mechanistic insight into the pathogenesis of adenomyosis.

## 1 Introduction

Adenomyosis is a common benign gynecological disease and a serious global health burden, characterized by the growth of endometrial tissue within the muscular layer of the uterus (1). Women with adenomyosis suffer from a series of symptoms, such as dysmenorrhea and abnormal uterine bleeding, and are at increased risk of infertility (2). The initiation and progression of adenomyosis constitute a series of complex biological processes involving multiple molecular dysregulations. It has long been hypothesized that adenomyosis is a composite result of intrinsic factors (such as retrograde menstruation and coelomic metaplasia) and external injuries (3).

Recently, adenomyosis has been increasingly recognized as a disease with disordered immune balance at both systemic and local levels (4). The dysregulated immune cells exert their effect by direct cell-cell interactions and the secretion of cytokines and growth factors. This altered immune landscape significantly impacts the behavior of adjacent cells, driving the processes such as angiogenesis (5), and epithelial-mesenchymal transition (EMT) (6). Interaction of macrophages and endometrial cells induces epithelial-mesenchymal transition-like processes in adenomyosis (6), fibrosis formation (7), and ultimately creates a cycle of tissue damage and repair. While most studies focus on immune alteration within eutopic endometrium, the findings remain inconsistent and inconclusive. For instance, some studies reported variations in the number of natural killer cells within eutopic adenomyosis endometrium, while others indicated no significant changes (8–10). However, functional studies on the specific immune cell subtypes often yielded conflicting results and a systemic characterization of the uterine immune microenvironment underlying disease pathology is lacking.

Additionally, conflicting evidence also exists regarding cytokine profiles, where both pro-inflammatory and anti-inflammatory cytokines were found at increased levels within eutopic or ectopic endometrium (11–13). This paradoxical evidence underscores the intricate and dynamic nature of the local immune environment in adenomyosis, which can vary depending on the genetic differences and disease stages. On the other hand, these discrepancies are also likely attributable to the inaccuracy of traditional methods, such as quantitative reverse transcription polymerase chain reaction (qRT-PCR) and immunohistochemistry (IHC), which rely on the measurement of a limited number of immune cell marker genes. These approaches often fail to provide a comprehensive and reliable assessment of the immune landscape in adenomyosis. To address this gap, advanced high-throughput sequencing technologies, such as transcriptomics, are needed to investigate the molecular variations and immune alterations within adenomyosis.

Notably, recent emerging studies have revealed significant alterations in metabolic processes within uterine diseases, such as cervical cancer and endometriosis, offering new insights into the underlying pathogenesis (14, 15). However, the application of metabolomics to explore global metabolic variations associated with adenomyosis remains limited. One study assessing the serum metabolic profiles observed a distinct metabolic signature in individuals diagnosed with adenomyosis, characterized by lower concentrations of 3-hydroxybutyrate, glutamate and serine compared with controls (16). Another study identified numerous altered metabolites related to oxidative stress, inflammation, cell proliferation and energy production within the myometrium of women with adenomyosis, by gas chromatography coupled with mass spectrometry (GC-MS) and ultra-high performance liquid chromatography coupled with mass spectrometry (UHPLC-MS) (17).

Despite the fact that immune and metabolic dysregulation in adenomyosis has been studied individually, there is still a notable lack of research focus on the interplay between these two systems within eutopic adenomyosis endometrium. How metabolic changes influence immune responses—or vice versa—has yet to be fully elucidated. In oncology research, however, the interplay between immune system and metabolism have been well-documented (18). For instance, Leone et al. demonstrated that the blockade of glutamine could reshape cancer cell metabolic programs, thereby enhancing anti-tumor response (19). Similarly, Yan et al. showed that cholesterol deficiency can lead to T cell exhaustion and dysfunction in the cancer context, thus contributing to the progression of disease (20). Considering the insights from the tumor field and the central role of both immune and metabolic alterations, it is valuable to investigate the immune-metabolic crosstalk within the context of adenomyosis, a topic which is still under-explored.

In this study, we aimed to elucidate the disrupted immune landscape and metabolic microenvironment in the eutopic endometrium of adenomyosis, as well as the interplay between the two components. To achieve this goal, we analyzed microarray, bulk RNA-sequencing and single-cell RNA sequencing data using both in silico approaches and experimental validation. We specifically focused on M2 macrophages, keratan sulfate biosynthesis, and the key genes involved in this pathway. The current study provides novel insights into the immune-metabolic crosstalk in adenomyosis, highlighting potential molecular targets for therapeutic intervention.

## 2 Methods

### 2.1 Acquisition and processing of microarray and bulk RNA-seq data

**Figure 1:**
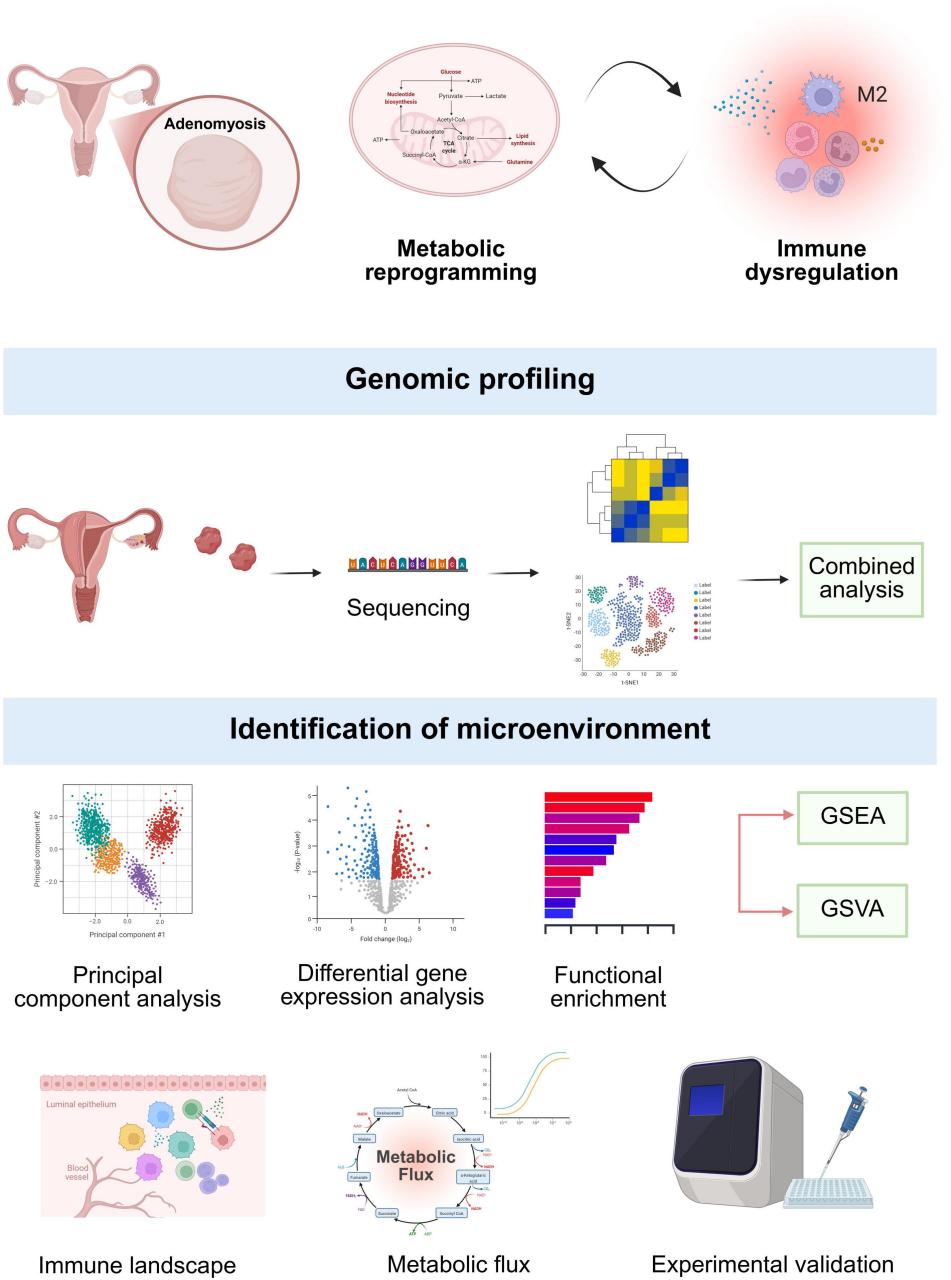
Schematic representation of this study. GSEA, gene set enrichment analysis; GSVA, gene set variation analysis.

The overall workflow of this study is depicted in **Figure 1**. The microarray dataset GSE78851 in CEL format was downloaded from GEO (https://www.ncbi.nlm.nih.gov/geo/). The microarray dataset comprises 3 eutopic endometrium biopsies (endometrium obtained from patients diagnosed with adenomyosis) and 5 normal endometrium biopsies (endometrium obtained from healthy women without adenomyosis). Raw CEL files were imported into the R and processed with the “oligo” (version 1.68.2) package, which supports various Affymetrix platforms. Background correction, normalization, and probe summarization was carried out using the Robust Multichip Average (RMA) method. The probe was annotated using the package “hugene10sttranscriptcluster.db” (version 8.8.0) to ensure accurate mapping to gene identifiers. Principal Component Analysis (PCA) was performed using the package “FactoMineR” (version 2.11), and visualized with “factoextra” (version 1.0.7). The RNA-seq data in fastq format of GSE193928 was downloaded from European Nucleotide Archive (https://www.ebi.ac.uk/ena/browser/home), followed by quantifying against the human reference transcriptome ENSEMBL v87 using Salmon (21). From the original dataset, only adenomyosis and control samples were included. Following quality control, four outliers were excluded, yielding a final dataset of 6 adenomyosis samples and 15 control samples.

### 2.2 Identification of DEGs and functional enrichment analysis

Differential expression analysis was performed with the “limma” (version 3.60.4) package, incorporating empirical Bayes moderation to enhance statistical robustness. Genes meeting criteria of |Fold Change| > 1.5 and p-value < 0.05 (Benjamini-Hochberg correction) were considered significant. To analyze the enriched pathways, we used the R package “clusterProfiler” (version 4.8.2) (22). For gene set enrichment analysis (GSEA), all detected genes were ranked according to Log2Fold Change in descending order and subsequently used as input. Pathway databases utilized included KEGG (23) and WikiPathways (24). Pathways with a p-value < 0.05 were regarded as significantly enriched.

### 2.3 Immune decomposition analysis

For CIBERSORT analysis, the R package “CIBERSORT” (version 0.1.0) was used to calculate the estimated immune cell proportion (25). The gene expression data was used as input with the LM22 signature matrix applied for cell-type deconvolution. For xCell analysis, the website tool xCell (https://comphealth.ucsf.edu/app/xcell) was used to deconvolute the immune and stromal cell composition of the endometrial samples based on gene expression data (26).

### 2.4 Gene set variation analysis

Gene Set Variation Analysis (GSVA) was performed to assess the variation in pathway activity across samples in the microarray data. The analysis was conducted using the R package “GSVA” (version 1.52.3) (27). KEGG pathway gene sets were retrieved using the KEGGREST (version 1.44.1) package, focusing on all human pathways with particular emphasis on metabolism pathways. Statistical comparisons between adenomyosis and control groups were conducted using Student’s t-tests for individual pathways. Statistical significance was defined as p < 0.05.

### 2.5 Acquisition and processing of single cell RNA-seq data

We obtained the annotated and precomputed (UMAP) endometrial scRNA-seq dataset from the web portal of Reproductive Cell Atlas (https://www.reproductivecellatlas.org/endometrium_reference.html), which contains about 314,000 cells from 63 donors (28). Then, we conducted quality control of the dataset, excluded the cells derived from the patients after taking hormones, irrelevant cell types (e.g., cells derived from the cervix, red blood cells). As a result, we got a dataset with a total of 238,211 cells and 17,736 genes. Further downstream analysis and visualization were conducted with Seurat workflow. Specifically, we visualized the dataset using the R package “dittoSeq” (version 1.14.3) and “scCustomize” (version 3.0.0).

### 2.6 METAFlux analysis

METAFlux analysis was conducted according to the standard pipeline using the R package “METAFlux” (version 0.0.0.9000) (29). Firstly, we calculate the metabolic reaction activity score for each sample, which was subsequently used as the input for flux calculation. The calculation of flux was conducted using human blood as the medium, providing a physiologically relevant environment for the metabolic reactions under investigation. For the single cell RNA-sequencing data, average expression level was calculated for each distinct cell type, and then used as input for determining metabolic reaction activity score. Relative changes in metabolic pathway activity were calculated, with pathways showing differences > 0.1 classified as upregulated and those with differences < -0.1 classified as downregulated.

### 2.7 RNA extraction and RT-qPCR

Endometrial biopsy samples from patients diagnosed with adenomyosis and healthy controls were collected using a Pipelle device after obtaining written informed consent from all participants. The study protocol was approved by the Ethics Committee of Ren Ji Hospital, Shanghai Jiao Tong University School of Medicine (Approval No. KY2021-211-B).

Total RNA was isolated from collected endometrial tissue biopsies using the FastPure Complex Tissue/Cell Total RNA Isolation Kit (Vazyme), following the manufacturer’s instructions. Complementary DNA (cDNA) was synthesized using the PrimeScript™ RT Master Mix (Takara). RT-qPCR was carried out using the Taq Pro Universal SYBR qPCR Master Mix (Vazyme) on Applied Biosystems QuantStudio™ 7 Flex. Forward and reverse primer sequences used for amplification are provided in **Supplementary Table 1**. β-actin (*ACTB*) and glyceraldehyde-3-phosphate dehydrogenase (*GAPDH*) were selected as endogenous reference genes. Relative expression levels were quantified using the 2^–ΔΔCt method.

### 2.8 Immunofluorescence staining analysis

Human endometrium tissue biopsies were fixed in 4% paraformaldehyde (Sangon Biotech), embedded in paraffin and sectioned for immunofluorescence staining. Briefly, sections were deparaffinized, rehydrated, and subjected to antigen retrieval in Tris/EDTA buffer (pH 9.0). After blocking with 5% goat serum, tissue sections were incubated overnight at 4°C with primary antibodies, including anti-CD163 (ab182422, Abcam; 1:500), and anti-B4GALT3 (11041-1-AP, Proteintech; 1:200). Following washes in PBST, HRP-conjugated secondary antibodies were applied. Fluorescence signal amplification was achieved using TSA reagents for 3 minutes, followed by PBST washes. Nuclei were counterstained with DAPI for 10 minutes. Sections were imaged using a 3DHISTECH Pannoramic 250 FLASH scanner.

### 2.9 Statistical analysis and scientific plotting

Data visualization, including volcano plots, PCA plots, boxplots and correlation plots, was generated using the “ggplot2” (version 3.5.1) packages. Heatmaps were created by “pheatmap” (version 1.0.12). GSEA plots were generated by the package “enrichplot” (version 1.24.2). Venn diagrams were constructed by the online tool (https://bioinformatics.psb.ugent.be/webtools/Venn/). The workflow and schematic diagram were created with BioRender.com. Bioinformatic analyses were performed in R version 4.4.0. RT-qPCR analysis was performed using Prism (v10.1.2). Statistical analyses were conducted using a two-tailed Student’s t-test to evaluate significant differences between two groups. One-way ANOVA was employed to assess differences among multiple groups. Statistical significance was defined as p < 0.05.

## 3 Results

### 3.1 Transcriptional changes in adenomyosis compared to normal control

To investigate the transcriptional differences between the endometrium from patients diagnosed with adenomyosis and that of normal controls, we analyzed the dataset GSE78851 retrieved from GEO (30). This dataset comprises eight endometrial samples, including 5 samples from healthy controls and 3 from patients diagnosed with adenomyosis. Our analysis began with an exploratory principal component analysis (PCA) to assess the overall transcriptional landscape of the samples. The PCA results revealed clear distinctions between the transcriptional profiles from eutopic endometrial samples and those from the normal endometrium samples (**Supplementary Figure 1A**). This separation underscores significant variances in gene expression pattern between the two groups, indicating the presence of disease-specific molecular signatures in adenomyosis.

**Figure 2:**
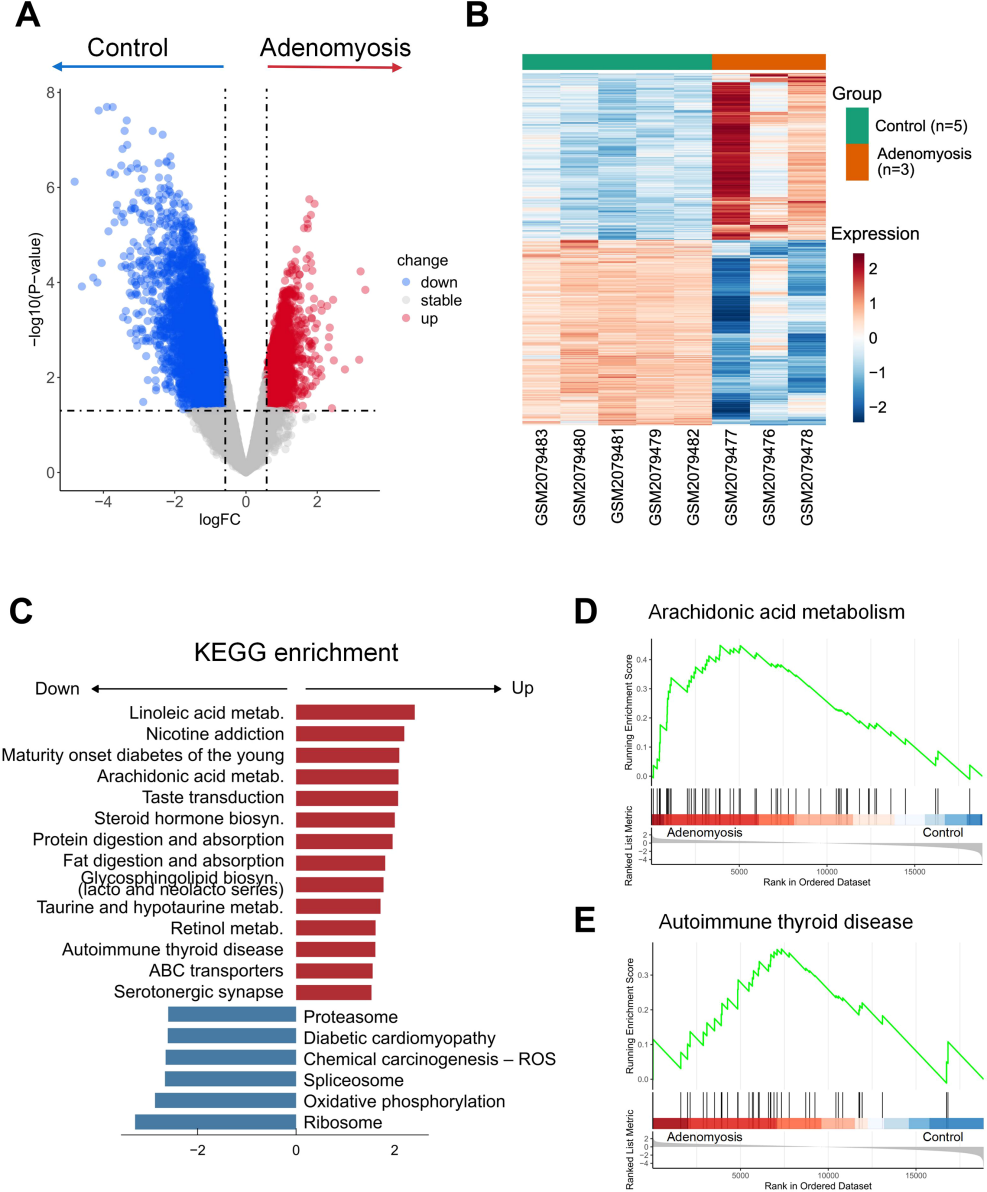
Transcriptomic alterations distinguish adenomyosis from normal control. **(A)** Volcano plot showing the differentially expressed genes between the eutopic and control endometrium from dataset GSE78851. A cut-off value of |Fold Change| > 1.5 and p-value < 0.05 was used as the threshold. Red and blue dots stand for the significantly upregulated and downregulated genes respectively, while the gray dots indicate non-significant genes. **(B)** Heatmap displaying the scaled expression level of the DEGs between eutopic and control endometrium. Each row stands for a DEG, and each column stands for a sample. **(C)** The bidirectional barplot showing GSEA results based on the KEGG database. The x-axis represents the normalized enrichment scores (NES). The positive value indicates that the pathways are enriched in the adenomyosis group, while the negative value indicates that the pathways are enriched in the control group. For all terms, p-value < 0.05. **(D, E)** GSEA plots displaying the enrichment of arachidonic acid metabolism **(D)**, and autoimmune thyroid disease **(E)**. The enrichment score indicates the pathway activity in the adenomyosis *versus* control.

To further investigate the transcriptional alterations associated with adenomyosis, we conducted comprehensive transcriptomic analysis. The genes meeting the criteria of |FoldChange| > 1.5 and p-value < 0.05 were identified as differentially expressed genes (DEGs). Specifically, we identified a total of 7,293 DEGs, including 3,452 upregulated genes and 3,841downregulated genes in adenomyosis group compared to the control group (**Figure 2A**, **Supplementary Table 2**). These findings demonstrate global transcriptional reprogramming associated with adenomyosis, reflecting profound molecular disruptions potentially linked to the pathology of the disease.

To validate the observed gene expression variances, we performed PCA analysis using only the identified DEGs. The refined analysis emphasized on the separation between adenomyosis and control samples, suggesting that these DEGs are key contributors to the distinct transcriptional landscape of adenomyosis (**Supplementary Figure 1B**). Subsequently, we carried out hierarchical clustering analysis of the DEGs to offer an additional layer of validation. This analysis confirmed the transcriptional divergence, with adenomyosis and control samples forming two distinct and non-overlapping clusters (**Figure 2B**).

### 3.2 Activated immune-related pathways in the eutopic endometrium of adenomyosis

To further explore the functional pathways potentially contributing to the pathogenesis of adenomyosis, we performed GSEA to evaluate the enrichment of predefined signaling pathways based on all detected genes (**Figure 2C**, **Supplementary Figure 1C**, **Supplementary Tables 3**, **4**). The results demonstrated strong positive enrichment of the pathway arachidonic acid metabolism in the adenomyosis group (**Figure 2D**). This signaling pathway has been recognized as a potent mediator of inflammation. It plays an essential role in the synthesis of downstream prostaglandins, which are key inflammatory factors causing, among others, the chronic symptoms of other endometrial diseases like endometriosis (31). These findings emphasize the critical role of immune-related processes in the pathophysiology of adenomyosis, consistent with the known inflammatory microenvironment of the disease.

Additionally, we also uncovered novel pathways associated with immune response, including autoimmune thyroid disease (**Figure 2E**), overview of proinflammatory and profibrotic mediators (**Supplementary Figure 1D**), eicosanoid synthesis (**Supplementary Figure 1E**). Actually, eicosanoid synthesis is a pathway in the downstream of arachidonic acid metabolism, where the enzyme cyclooxygenase converts arachidonic acid into various bioactive lipid molecules called eicosanoids (32), including prostaglandins. This outcome thus provides more details about the downstream signaling of the identified pathway arachidonic acid metabolism, indicating eicosanoids might be the potential contributing factors of adenomyosis. Moreover, the enrichment of profibrotic mediators could be an explanation of the excessive fibrosis found within the eutopic endometrium, which has been recognized as a feature of adenomyosis (33). Together, these results highlight immune dysregulation as a key molecular hallmark of adenomyosis.

### 3.3 Disrupted immune microenvironment in adenomyosis

**Figure 3:**
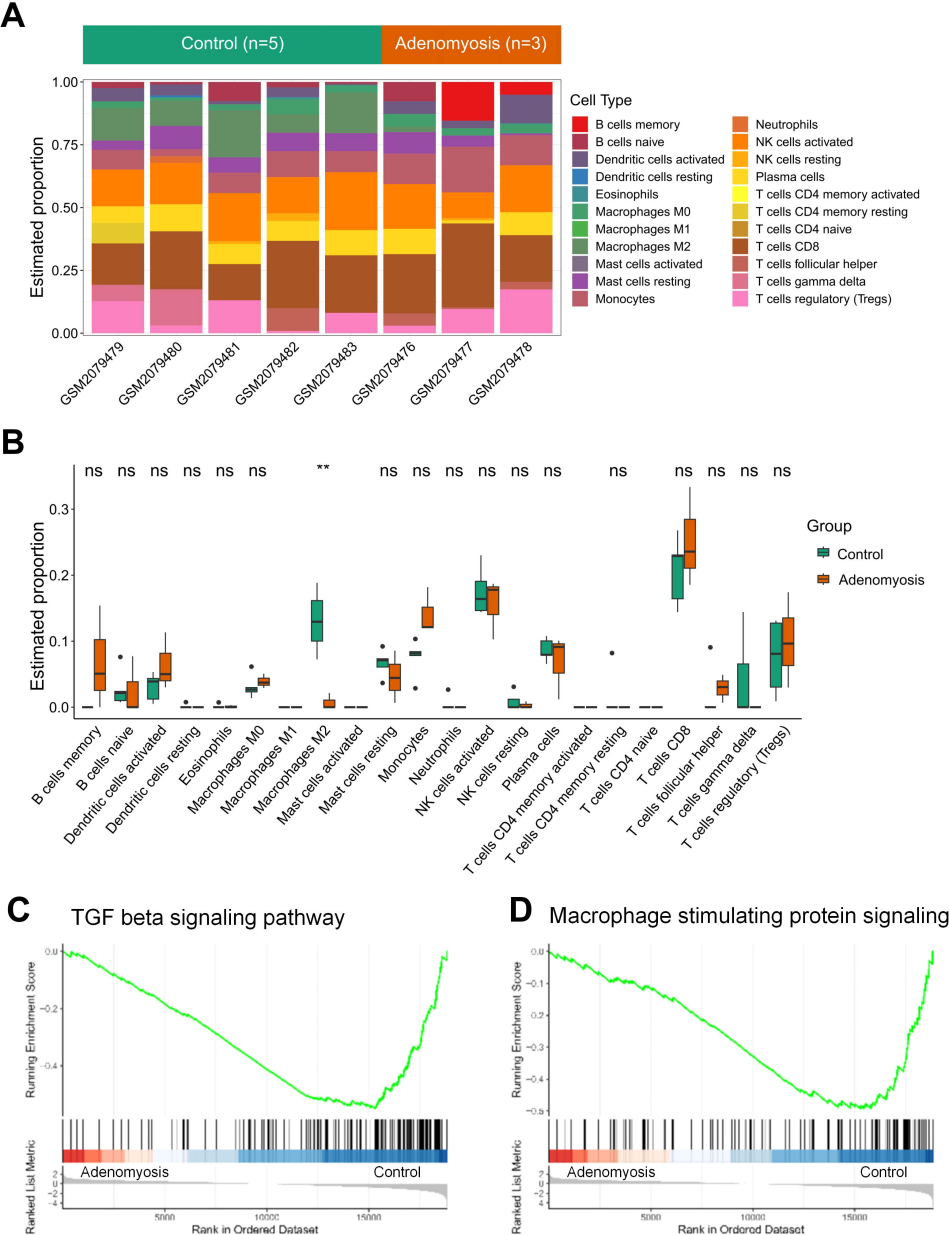
Transcriptomic analyses characterize immune infiltration patterns in adenomyosis. **(A)** Stacked bar plot showing the estimated proportions of 22 immune cells calculated by CIRBERSORT in eutopic and control endometrium samples based on dataset GSE78851. **(B)** Boxplot comparing the immune cell proportions between eutopic and control endometrium based on CIBERSORT analysis. Student’s t-test was used to calculate the significant changes between groups, with double asterisks (**) indicating p-value < 0.01 and “ns” standing for no significant difference between two groups. **(C, D)** GSEA plots showing the downregulation of TGF-beta signaling pathway **(C)** and Macrophage Stimulating Protein (MSP) signaling pathway **(D)** in the adenomyosis group.

To comprehensively explore the immune status of the eutopic endometrium, we applied comparative analysis of immune infiltration in eutopic versus healthy endometrium, based on CIBERSORT (25) and xCell (26). Compared with normal controls, the adenomyosis group exhibited a distinct immune signature, suggesting an altered immune landscape within the endometrium from patients diagnosed with adenomyosis (**Figure 3A**).

Significant changes were manifested in specific immune cell subsets between the adenomyosis and control groups (**Figure 3B**). Notably, M2 macrophages, known for their anti-inflammatory and tissue-remodeling roles (34), showed a marked decrease in adenomyosis samples, indicating an overall inflammatory shift within eutopic endometrium. Immune cell deconvolution with xCell revealed a significantly decreased infiltration score of macrophages in eutopic endometrium, which might result from the reduced number of M2 macrophages (**Supplementary Figure 2A**). To strengthen the reliability of the findings, we extended the CIBERSORT analysis to an independent dataset GSE193928 (**Supplementary Figure 2B**). The result consistently demonstrated a significant reduction of M2 macrophage abundance in the adenomyosis group, reinforcing the notion that the disrupted M2 macrophage is a key feature of this disease. This finding aligns with our previous functional enrichment analysis, which pointed to signaling pathways associated with inflammation as potential contributors of adenomyosis. This disordered immune profile, characterized by heightened inflammatory status, may drive abnormal cell proliferation and invasion of eutopic endometrial tissue and the persistence of chronic pain caused by adenomyotic lesion.

Interestingly, we also observed a marked downregulation of key pathways involved in promoting M2 macrophage polarization in the adenomyosis group compared to the control group (**Figures 3C, D**). Specifically, the TGF beta signaling pathway and macrophage stimulating protein (MSP) signaling were significantly suppressed in the adenomyosis group. Both TGF beta and macrophage stimulating protein are critical regulators for M2-like macrophage polarization and play essential roles in fostering the anti-inflammatory phenotype characteristic of M2 macrophages (35, 36). The reduced activity of these pathways may contribute to the diminished presence of M2 macrophages observed in the adenomyosis group, further reinforcing the inflammatory shift observed in eutopic endometrium.

### 3.4 Metabolic reprogramming in adenomyosis revealed by GSVA and METAFlux analysis

**Figure 4:**
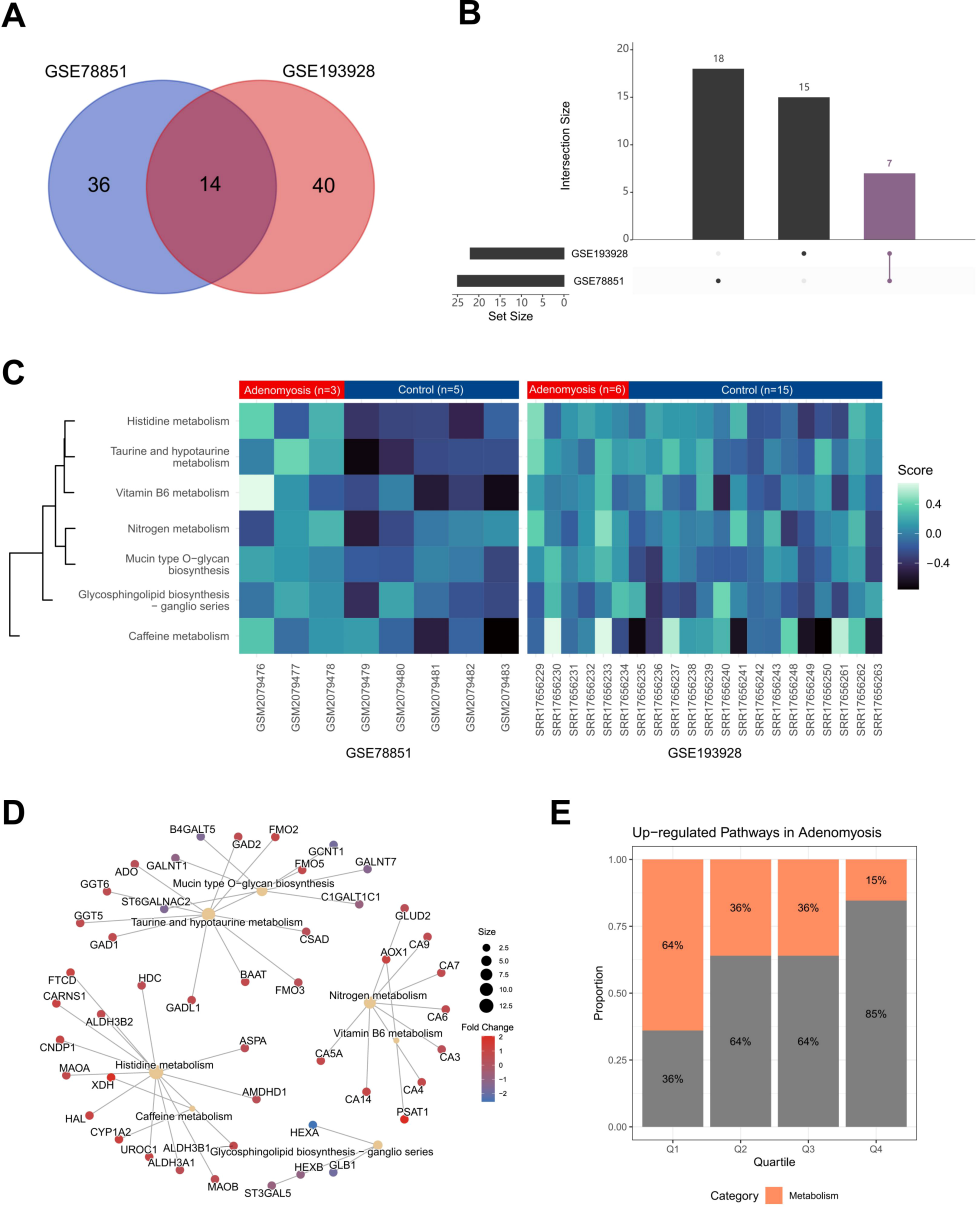
GSVA analysis identifies dysregulated metabolic pathways in adenomyosis across independent datasets. **(A)** Venn diagram showing the overlap of upregulated pathways identified by GSVA in GSE78851 (n = 50) and GSE193928 (n = 54), with 14 pathways commonly enriched in both datasets. **(B)** Upset plot illustrating dataset-specific and shared metabolism-related pathways, with 18 unique to GSE193928, 15 unique to GSE78851, and 7 commonly enriched in both datasets. **(C)** Heatmap showing GSVA enrichment scores of the seven commonly enriched metabolism-related pathways across individual endometrial samples from datasets GSE78851 and GSE193928. Color intensity represents scaled GSVA scores, with each row corresponding to a pathway and each column representing a sample. **(D)** Visual representation of seven significantly altered metabolism-related pathways and their constituent genes. Nodes represent pathways, with edges indicating pathway-gene associations. Node size corresponds to the number of connections, and color intensity reflects the fold change difference between the eutopic endometrium relative to the healthy control endometrium. **(E)** Proportion of metabolism-related pathways among all activated pathways. Pathways were ranked in descending order based on GSVA enrichment scores and divided into four quartiles (Q1–Q4). The x-axis represents the quartiles, and the y-axis shows the proportion of metabolism-related pathways within each quartile.

To investigate the molecular alterations underlying adenomyosis while taking patients variability into consideration, we performed GSVA based on KEGG database and attempted to find commonly activated pathways across different adenomyosis datasets. Unlike GSEA and over-representation analysis (ORA), GSVA provides sample-level enrichment scores, allowing us to capture pathway activity variations across individuals (27). Using this approach, we identified 50 pathways upregulated in GSE78851 and 54 in GSE193928, with 14 pathways commonly activated in both datasets (**Figure 4A**). This supports the existence of divergent pathways activation pattern that might contribute to the heterogeneity of the disease in addition to the shared biological processes.

Given the central role of metabolism in cellular physiology and its emerging relevance in benign gynecological disorders, we examined metabolism-associated pathways. Of the identified pathways, 18 were unique to GSE193928 and 15 unique to GSE78851, while 7 were shared between both datasets (**Figure 4B**). Notably, half of the 14 shared pathways were metabolism-related (**Figures 4B, C**), including nitrogen metabolism, taurine and hypotaurine metabolism, glycosphingolipid biosynthesis - ganglio series, mucin type O-glycan biosynthesis, vitamin B6 metabolism, caffeine metabolism, histidine metabolism. Several of these metabolic processes have been previously reported as disrupted in gynecological conditions. For example, nitrogen metabolism is significantly altered in the endometrium of nonhuman primates with endometriosis (37), and taurine deficiency impairs uterine receptivity and embryo implantation in mice (38), underscoring their potential relevance to endometrial microenvironment.

To further dissect the shared metabolic alterations, we mapped the seven commonly enriched metabolism-related pathways and their constituent genes (**Figure 4D**). Interestingly, the pathways were composed of distinct gene sets without apparent overlap, indicating adenomyosis is associated with broad and multifaceted metabolic perturbations, rather than being driven by a single core metabolic axis. Such separation underscores the complexity of the disease, where multiple distinct metabolic programs are simultaneously reprogrammed. Furthermore, pathway activity ranking demonstrated that nearly two-thirds of the most highly activated pathways were metabolism-related (**Figure 4E**). This predominance highlights the central role of metabolic alterations in shaping the pathogenesis of adenomyosis and suggests that alterations in diverse metabolic processes may collectively shape its pathogenesis.

**Figure 5:**
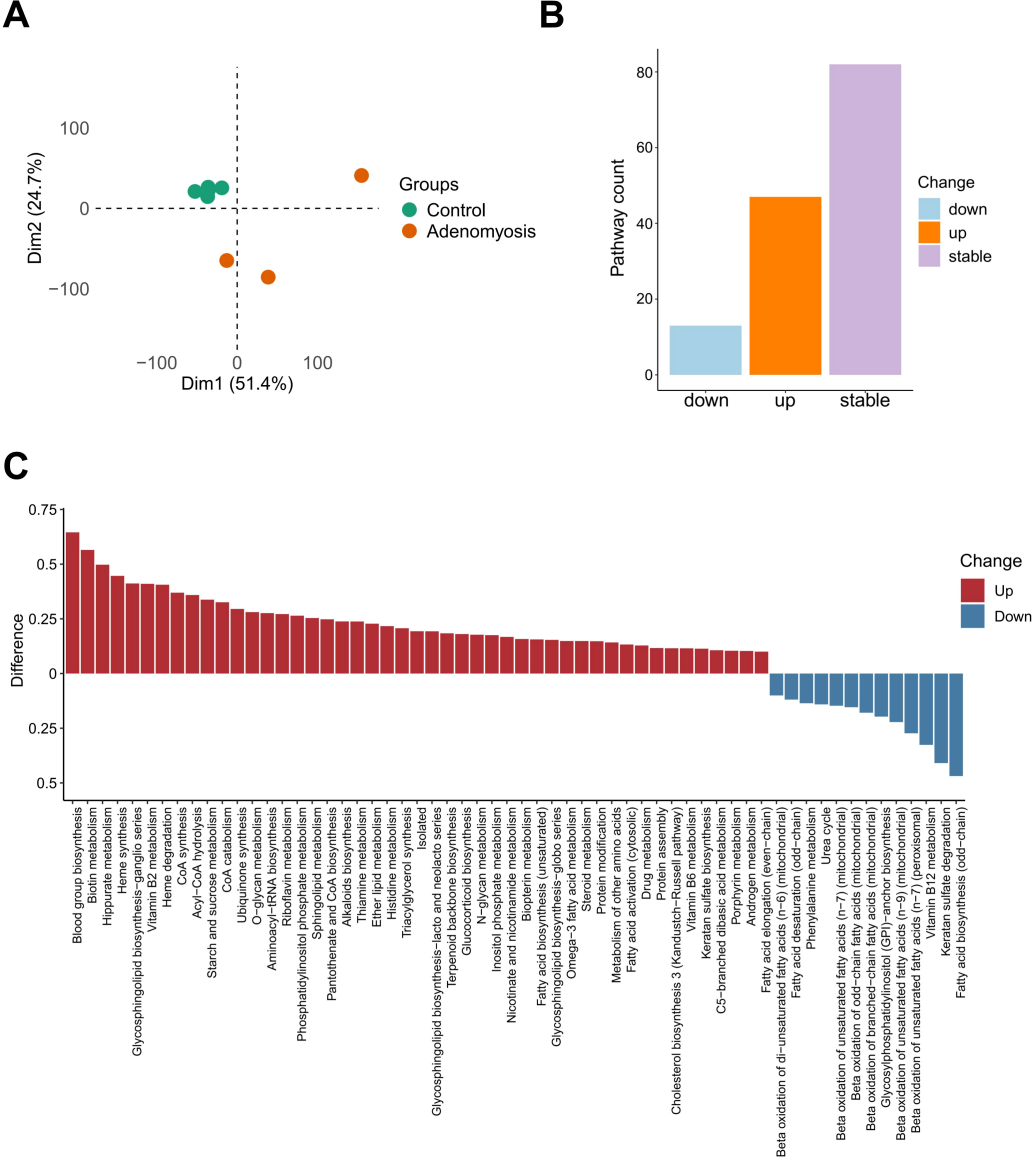
Flux analysis reveals metabolic reprogramming underlying adenomyosis. **(A)** PCA plot showing distinct clustering of endometrial samples based on metabolic reaction activity score between control (green) and adenomyosis (orange) group. **(B)** Barplot illustrating the number of metabolic pathways identified as downregulated, upregulated, or stable between adenomyosis and control samples. **(C)** Waterfall plot of differentially regulated metabolic pathways. Pathways with increased activity in adenomyosis compared to controls are shown in red, and those with decreased activity are shown in blue.

Despite the promising preliminary findings based on gene expression data, the specific metabolic landscape within eutopic endometrium remains poorly defined due to the inherent discrepancies between gene expression levels and actual metabolite production. These limitations partly arise from intricate post-transcriptional and post-translational regulations (39), which can significantly affect the activity of enzymes, thereby altering the efficiency of metabolic reactions. To overcome this limitation and address the gap in knowledge, we utilized METAFlux to characterize the metabolic shifts in adenomyosis that may contribute to the disease pathogenesis. METAFlux analysis was carried out according to the standard workflow outlined in the Methods section. Through clustering of metabolic reaction activity scores, we were able to distinguish two clusters of endometrium samples (i.e. control and adenomyosis; **Figure 5A**). This finding further confirmed the metabolic divergence between adenomyosis and control group and underscored the metabolic reprogramming in the pathogenesis of adenomyosis. To identify the most disrupted metabolic pathways, we quantified the differences in metabolic pathway activities. Specifically, the pathways exhibiting a difference > 0.1 were considered as upregulated, while those with a difference < -0.1 were recognized as downregulated. In total, we identified 47 upregulated and 13 downregulated pathways (**Figure 5B**, **Supplementary Table 5**), shedding light on the key metabolic alterations associated with adenomyosis. The distribution of pathway activity differences further highlighted key metabolic shifts, with some pathways significantly altered within eutopic endometrium (**Figure 5C**).

Among the altered pathways detected by METAFlux, histidine metabolism (**Supplementary Figure 3A**) was also identified at the transcriptomic level, consistent with GSVA results (**Figure 4C**), reinforcing its potential role in the context of eutopic endometrium. Additionally, we also identified a series of metabolic disorders which cannot be obtained from the gene expression level (**Figure 5C**, **Supplementary Figures 3B–F**), such as hippurate metabolism, fatty acid biosynthesis, ubiquinone synthesis and propanoate metabolism, and riboflavin metabolism. These findings underscore the importance of integrating METAFlux analysis with gene expression profiling to gain a more comprehensive understanding of metabolic landscape and disease mechanisms.

### 3.5 Crosstalk between immune infiltration and altered metabolic processes

**Figure 6:**
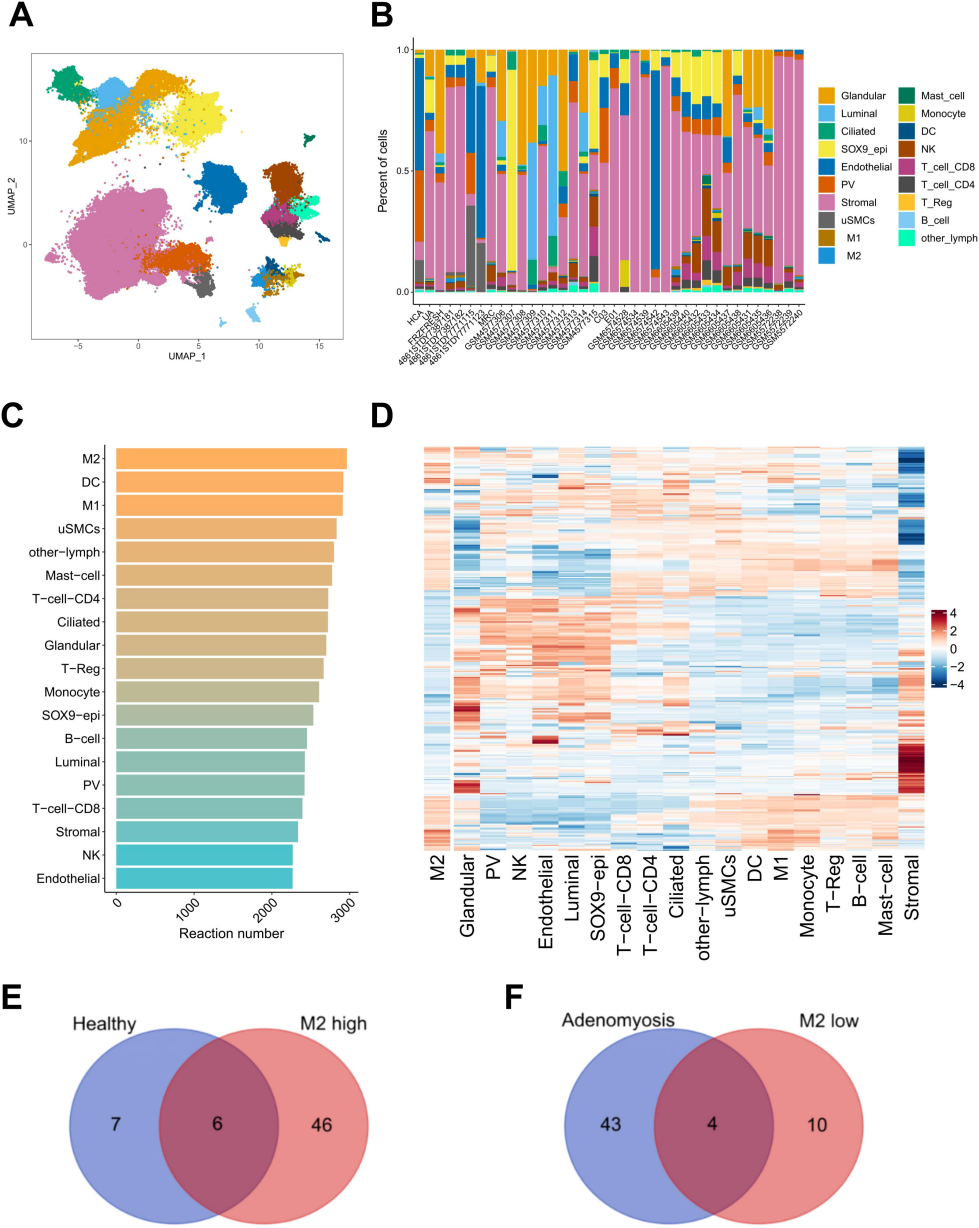
Crosstalk between immune infiltration and altered metabolic processes. **(A)** UMAP visualizing different cell types identified in the single cell RNA-sequencing dataset. **(B)** Stacked barplot showing the proportional composition of various cell types across individual samples involved in the single cell RNA-sequencing dataset. The colors correspond to the cell types identified in **(A)**. **(C)** Barplot summarizing the number of metabolic reactions of different cell types based on the top 5% of metabolic reactions with the highest or lowest flux scores. The color intensity indicates the number of reactions. **(D)** Heatmap depicting the activity of the most active metabolic reactions in the M2 macrophages across different cell types. The color intensity indicating the scaled flux score calculated by METAFlux analysis. **(E)** Venn diagram depicting overlap between signatures of endometrium from healthy donors and M2-high. **(F)** Venn diagram depicting overlap between signatures of endometrium from donors with adenomyosis and M2-low.

To further decipher the complex relationship between immune dynamics and metabolic processes at single cell level, we extended our analysis to a single cell RNA-seq dataset to explore the cell type-specific metabolic features within endometrium. In order to obtain a more comprehensive understanding of both the healthy and the diseased states of endometrium, we employed a dataset comprising endometrial biopsies from women with or without benign gynecological diseases (28). Following rigorous quality control procedures, we excluded unwanted cells, including those derived from hormone-treated patients, cells derived from the cervix, as well as irrelevant cell types (e.g., red blood cells), resulting in a dataset of 238,211 cells and 17,736 genes. The endometrial cells were subsequently clustered into four major subtypes, including epithelial cells (glandular, luminal, ciliated and SOX9+ epithelial cells), endothelial cells, mesenchymal cells (stromal cells, smooth muscle cells, perivascular cells), and immune cells (M1, M2, mast cells, monocytes, dendritic cells, natural killer cells, CD8+ T cells, CD4+ T cells, Treg, B cells, other lymphocytes) (**Figure 6A**). Feature plots illustrating the expression of canonical marker genes defining these identities are provided (**Supplementary Figure 4**). Importantly, a cell composition variance was observed among samples from different patients, reflecting individual variability within the endometrial tissue (**Figure 6B**). This heterogeneity underlines the complexity of the endometrial environment, which requires precise regulation of both immune responses and metabolic processes.

To explore the metabolic heterogeneity of the identified cell types, we conducted METAFlux analysis to infer the metabolic activity of predefined processes at a cell type-specific resolution. The bar plot summarizes the metabolic activity of various cell types, as assessed using the top 5% of metabolic reactions with the highest or lowest flux score (**Figure 6C**). M2 macrophages, DC and M1 macrophages display the highest number of highly active reactions, indicating their heightened metabolic activity compared to other cell types. This finding again underscores the essential role of M2 macrophages in maintaining the metabolic homeostasis within endometrial microenvironment. To delve deeper into the metabolic characteristics of M2 and their metabolic interplay with other cell types, we selected and visualized the most active reactions identified in M2 macrophages. The heatmap reveals that M2 share complementary metabolic activity with stromal cells and glandular epithelial cells in some key reactions (**Figure 6D**), suggesting coordinated metabolic interactions essential for maintaining endometrial homeostasis.

Given the active and central role of M2 macrophages in the endometrial microenvironment, where they play a dual role in immune and metabolic homeostasis, we further investigated the relationship between metabolic activity and M2 abundance (**Supplementary Table 6**). Among all metabolic pathways, 52 showed a positive correlation with M2 abundance, potentially playing a critical role in promoting M2 polarization or proliferation (**Figure 6E**). Of these, six pathways were predominantly associated with healthy controls, highlighting their importance in maintaining normal endometrial function. Conversely, M2 infiltration was negatively correlated with 14 metabolic pathways, four of which were highly expressed in the eutopic endometrium, implicating these pathways in disease pathogenesis and their possible role in suppressing M2 macrophages (**Figure 6F**).

**Figure 7:**
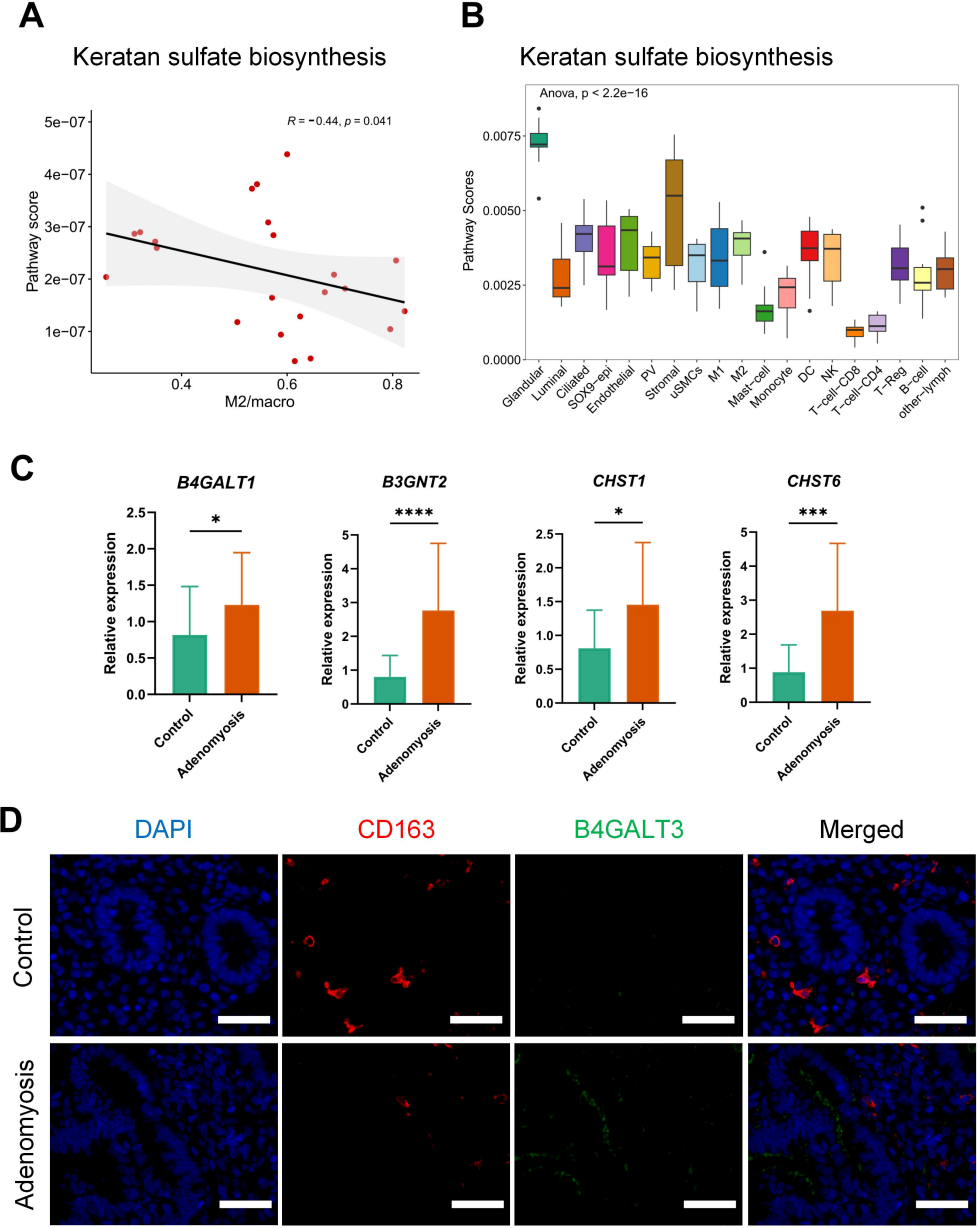
Activation of keratan sulfate biosynthesis in endometrial glandular epithelial cells from adenomyosis patients. **(A)** Correlation between the activity of keratan sulfate biosynthesis and M2 macrophage percentage calculated by Spearman correlation analysis. The y-axis represents the pathway score calculated by METAFlux, and the x-axis indicates the proportion of M2 macrophages among total macrophages. The correlation coefficient (R) and p-value are shown on the plot. **(B)** Boxplot illustrating the activity of keratan sulfate biosynthesis across different cell types. The y-axis represents pathway score, and the x-axis denotes the cell types. Statistical significance was assessed using one-way ANOVA. **(C)** Relative expression levels of *B4GALT1*, *B3GNT2*, *CHST1*, and *CHST6* in endometrium biopsies from adenomyosis patients and healthy controls, with *GAPDH* and *ACTB* as the reference genes. Intergroup comparisons were performed using a Student’s t-test. Data are presented as mean ± SD (n = 6 per group), with significance indicated by asterisks (*p < 0.05, **p < 0.01, ***p < 0.001, ****p < 0.0001). **(D)** Representative immunofluorescence images of CD163 and B4GALT3 co-staining in endometrial tissue from healthy controls (top) and adenomyosis patients (bottom). Scale bar: 50 µM. The blue color stands for DAPI (nuclei), red represents CD163, and green represents B4GALT3. The merged image shows the overlay of all three channels.

Among these, keratan sulfate biosynthesis emerged as significantly associated with M2 macrophages. Pathway activity analysis revealed an inverse correlation between keratan sulfate biosynthesis activity and M2 macrophage abundance (**Figure 7A**), raising the possibility that excessive keratan sulfate impairs M2 polarization or proliferation. As a key component of extracellular matrix, keratan sulfate plays a critical role in maintaining the normal function of the endometrium (40). Dysregulation of its biosynthetic pathway may therefore disrupt the delicate balance between extracellular matrix remodeling and immune cell regulation.

Further analysis revealed striking cell type-specific differences in keratan sulfate biosynthesis, with the highest activity in glandular epithelial cells (**Figure 7B**). A closer examination of the individual enzymatic reactions involved in this biological process revealed that each reaction exhibited maximal activity in glandular epithelial cells (**Supplementary Figure 5**). This consistency across both pathway-level and reaction-level analyses highlights the central role of glandular epithelial cells as the predominant source of keratan sulfate within the endometrial microenvironment, which may act as potential regulators of macrophages in the endometrial niche.

To further validate these observations at the experimental level, we performed RT-qPCR analysis on key genes involved in keratan sulfate biosynthesis, including *B4GALT1* (beta-1,4-galactosyltransferase 1), *B3GNT2* (UDP-GlcNAc:betaGal beta-1,3-N-acetylglucosaminyltransferase 2), *CHST1* (carbohydrate sulfotransferase 1), and *CHST6* (carbohydrate sulfotransferase 6), respectively. All four genes were significantly upregulated in eutopic endometrial biopsies from adenomyosis patients compared to healthy controls (**Figure 7C**), confirming the computationally predicted activation of keratan sulfate biosynthesis in disease tissue.

Furthermore, to strengthen the findings, we conducted immunofluorescence co-staining for CD163 (a marker of M2 macrophages) and B4GALT3 (beta-1,4-galactosyltransferase 3, one of the enzymes critical for keratan sulfate chain elongation). Notably, B4GALT3 was robustly expressed in the eutopic endometrium of adenomyosis patients but was almost undetectable in healthy endometrium (**Figure 7D**). Moreover, M2 macrophages were frequently localized around the glandular structures, where B4GALT3 expression was enriched, suggesting a potential spatial relationship between epithelial keratan sulfate production and M2 macrophage abundance.

Considering the fact that keratan sulfate homeostasis is regulated by both biosynthesis and degradation processes, we further investigate the degradation process within the endometrium. Unlike the biosynthesis process, degradation is not directly associated with M2 abundance (**Supplementary Figure 6A**), suggesting that other regulatory mechanisms may influence this process. However, keratan sulfate degradation exhibits a distinct cell type-specific pattern, with endometrial stromal cells, M2 macrophages, CD4+ and CD8+ T cells exhibiting a relatively high activity (**Supplementary Figures 6B, C**). Further analysis of metabolite flux confirmed the complementary roles of different cell types: epithelial cells and stromal cells actively release keratan sulfate I and II, while M2, mast cells and T cells predominantly uptake and degrade these products (**Supplementary Figure 6D**). These results collectively suggest that keratan sulfate homeostasis, maintained through the coordinated synthesis and degradation by various cell types, plays a critical role in modulating the endometrial microenvironment and is closely linked to the function of M2 macrophages.

## 4 Discussion

Adenomyosis is a challenging gynecological disorder with the outgrowth of endometrium tissue into the muscular layer of the uterus. Despite its prevalence in women of reproductive age, it has been under-explored for decades. Although significant advance has been made to decipher the hormonal imbalance underlying this condition, less is known about the molecular mechanisms that contributing to disease pathogenesis, particularly with respect to dysregulated immune system and altered metabolic pathways. In our study, we combined transcriptomic profiling with metabolic flux analysis, and uncovered distinct alterations in immune and metabolic pathways within the eutopic endometrium. Our key computational findings were further supported by experimental validation, underscoring the potential involvement of keratan sulfate biosynthesis and suggesting potential therapeutic targets for intervention.

Through comprehensive analysis, we revealed an imbalanced immune microenvironment within the eutopic endometrium, including enhanced immune-related signaling and altered immune cell abundance. Previous researches have proposed that the eutopic endometrium skewed to be an immune dysregulated status, with altered immune cell populations and imbalanced pro-inflammatory and anti-proinflammatory cytokines (4). Notably, our findings further demonstrated activation of arachidonic acid metabolism and upregulation of pro-inflammatory and pro-fibrotic mediators within the eutopic endometrium, suggesting indicating enhanced cellular activation that may be potentially involved in this condition. The main route of arachidonic acid metabolism is COX (cyclooxygenase) pathway, leading to the synthesis of various bioactive prostanoids, including prostaglandins (PGs), thromboxanes (TXs), and prostacyclin (PGI2) (41). These metabolites play a critical role in inflammatory response, which has been identified within the ectopic endometrium of endometriosis (31). Our evidence indicates that arachidonic acid metabolism and its downstream metabolites might be a contributor for the inflammatory status of adenomyosis. Importantly, its involvement indicates the shared etiological and pathological mechanisms between adenomyosis and endometriosis, both of which have been characterized by excessive inflammatory responses.

Previous studies declared variations of macrophage abundance in the eutopic and ectopic endometrium compared to the normal endometrium, as indicated by the expression level of the marker genes such as CD68 or CD163 (6, 9, 42). Our comparative analysis of immune landscape further revealed a significant decrease of M2 macrophages within the eutopic endometrium when compared to healthy endometrium. M2 macrophage is known for its anti-inflammatory role (43, 44), and its reduction provide extra explanation for the inflammatory shift observed within eutopic endometrium. The reduction in immune regulatory cells like M2 and the simultaneous increase in pro-inflammatory mediators work together to create a persistent chronic inflammatory microenvironment of adenomyosis. Notably, we find that the reduction in M2 macrophage abundance may be attributed to the suppression of key signaling pathways involved in M2 polarization, such as the TGF-beta signaling pathway and macrophage-stimulating protein (MSP) signaling. Both TGF-β and MSP play essential roles in activating macrophages, controlling their inflammatory response and promoting the production of cytokines (45, 46). Our findings provide novel insights into the dysregulated immune microenvironment within eutopic endometrium.

In addition to dysregulated immune system, our study uniquely shed light on the metabolic reprogramming that occurs in the eutopic endometrium. While the close interplay between metabolic regulation and immune cell function has been well-documented in the tumor microenvironment, where metabolic disturbances can profoundly influence macrophage function, polarization, and tumor progression (47), similar investigations in adenomyosis remain limited. To date, gene expression-based metabolic analysis has been widely applied to decipher the metabolic landscape, including the methods like GSEA (48), GSVA (27). However, these methods become particularly unstable in metabolic reactions governed by a small number of genes, typically fewer than ten (48). Meanwhile, this technique is also limited to enzymatic reactions. Thus, we utilized a more advanced tool METAFlux to comprehensively decipher the metabolic features within endometrium. METAFlux provides an *in situ* high-throughput measurement of metabolites and can estimate the metabolic flux from both bulk and single cell RNA-seq data (29). In addition to the gene expression-based metabolic shifts, we also detected extra metabolic disruptions which cannot be captured at the transcriptomic level. These findings underscore the importance of utilizing complementary techniques to build a more comprehensive conception of the metabolic landscape within eutopic endometrium. Moreover, we stress that metabolic reprogramming is an integral component of adenomyosis pathogenesis.

One of the most important findings of our study is the discovery of a potential crosstalk between immune dynamics and metabolic processes in the endometrium. Our combined analysis of single-cell RNA-seq data and METAFlux revealed complementary metabolic flux between different cell types within the endometrium, particularly between M2 macrophages and other non-immune cells, including stromal cells, glandular epithelial cells, indicative of metabolic crosstalk among these cells. Although distinct metabolic signature has been mentioned in some uterine disease at a gene expression level through single cell RNA-sequencing, such as endometriosis (49), recurrent implantation failure (50), few researches have explored the dynamic metabolic flux among difference cell types. Our findings underscore the importance of cell-cell interaction through metabolic flux in sustaining a normal microenvironment, highlight a novel aspect of cellular communication that has not been extensively studied.

Further investigation into the metabolic pathways associated with M2 macrophage revealed an inverse correlation between keratan sulfate biosynthesis and the abundance of M2 macrophages, suggesting a novel mechanism by which metabolic remodeling may modulate the endometrial immune microenvironment in adenomyosis. Keratan sulfate, a major component of the extracellular matrix, plays a critical role in maintaining endometrial structure and function (51, 52). In the eutopic endometrium from adenomyosis patients, we observed a metabolic imbalance characterized by increased keratan sulfate biosynthesis and reduced degradation, which may compromise ECM integrity and hinder effective immune cell activity, particularly that of M2 macrophages. The close spatial relationship between M2 macrophages and epithelial regions enriched for keratan sulfate related gene expression further supports the concept of localized immune-metabolic crosstalk.

At the molecular level, several enzymes involved in keratan sulfate biosynthesis were consistently upregulated in eutopic endometrium from adenomyosis patients, including *B4GALT1*, *B3GNT2*, *CHST1*, and *CHST6*. These findings implicate not only pathway-level disruption of ECM metabolism but also specific molecular drivers that could serve as therapeutic targets. For instance, *B4GALT1* has been shown to enhances N-linked glycosylation of PD-L1, thereby stabilizing PD-L1 and promoting immune evasion in lung adenocarcinoma (53), suggesting similar glycan-mediated immunomodulation in adenomyosis. Likewise, B3GNT2 has been shown to impair cell-cell interaction and reduce T cell activation in tumor (54), raising the possibility of a potential role in dampening immune surveillance in adenomyosis. CHST1, another enzyme elevated in our cohort, has been demonstrated to promote immunosuppressive signaling by enhancing Siglec binding to sialoglycans (55). The *CHST6* gene, known for its role in keratan sulfate sulfation, has been implicated in immune regulation through its involvement in pyroptosis, a form of inflammatory cell death (56). Its dysregulation may contribute to heightened inflammatory responses, suggesting a potential link to immune dysfunction in adenomyosis. These insights into the immune-metabolic crosstalk within the endometrial microenvironment offer novel ideas for the pathogenesis of adenomyosis and suggest potential therapeutic avenues for restoring homeostasis and halting disease progression.

Our study offers several advantages in advancing our understanding of adenomyosis. First, the utilization of METAFlux analysis allowed for the in-depth investigation of metabolic reprogramming within the endometrial tissue, which sketches the dynamic flow of metabolites through various metabolic pathways instead of a static snap shot of the traditional transcriptomic or proteomic analysis. Second, the parallel analysis of metabolic activity and immune landscape added a novel perspective on the pathogenesis of adenomyosis, a field that has remained largely under-explored for decades. Third, combined analysis of microarray, bulk RNA-seq and single-cell RNA-seq data provides both a general and a high-resolution view of the microenvironment of endometrium, identifying specific immune subset involved in disease pathophysiology. Finally, we strengthened the robustness of our findings through experimental validation, including RT-qPCR and immunofluorescence staining, which confirmed key molecular alterations at the tissue level.

There are several limitations to this study. For example, the relatively small sample size of the microarray dataset GSE78851 (3 adenomyosis *versus* 5 control samples) may introduce bias from inter-individual variability and limit statistical robustness. Currently, no large datasets are available to address this limitation. This dataset includes only samples collected during the proliferative phase of the menstrual cycle, thereby minimizing variability related to cycle stage, potentially enhancing the generalizability of our results. Moreover, the clear separation observed between the adenomyosis and control groups demonstrates the reliability and robustness of the dataset. Additionally, while traditional transcriptomic analysis provides valuable insights into gene expression levels, it has limitations in fully capturing the metabolic processes occur within the tissue due to the influences of post-transcriptional and post-translational regulation. By combining transcriptomic analysis with METAFlux analysis, we ensure a more accurate and comprehensive picture of the metabolic processes within the endometrium tissue. Finally, while our study identifies promising therapeutic targets related to keratan sulfate dysregulation through both in silico and experimental approaches, further mechanistic studies are required to elucidate the precise molecular pathways and their functional impact, which is essential for translating these findings into feasible clinical applications for patients with adenomyosis.

## 5 Conclusions

In general, our study provides a comprehensive characterization of the immune and metabolic alterations in the eutopic endometrium of adenomyosis patients. By combining transcriptomic profiling with metabolic flux analysis, we identified an imbalanced immune microenvironment, and altered keratan sulfate biosynthesis, highlighting novel immune-metabolic crosstalk. Experimental validation further confirmed key molecular changes, reinforcing the robustness of our findings. Targeting this imbalance, for example by inhibiting keratan sulfate synthesis through *B4GALT1*, *B3GNT2*, *CHST1*, and *CHST6*, could potentially help modulate the metabolic crosstalk and the immune response within the eutopic endometrium.

## Data Availability

Publicly available datasets were analyzed in this study. This data can be found here: GEO (https://www.ncbi.nlm.nih.gov/geo/) and the web portal (https://www.reproductivecellatlas.org/endometrium_reference.html).

## Supplementary material

Supplementary Figure 1 **(A)** Principal component analysis of the microarray dataset GSE78851 based on all detected genes. **(B)** Principal component analysis of the microarrary dataset GSE78851 based on the identified DEGs. **(C)** The bidirectional barplot showing GSEA results based on the Wikipathways database. The x-axis represents the normalized enrichment scores (NES). The positive value indicates that the pathways are enriched in the adenomyosis group, while the negative value indicates that the pathways are enriched in the control group. **(D, E)** GSEA plots displaying the enrichment of overview of proinflammatory and profibrotic mediators **(D)**, eicosanoid synthesis **(E)**. The enrichment score indicates the pathway activity in the adenomyosis *versus* control.

Supplementary Figure 2 **(A)** Boxplot comparing the immune cell infiltration score between eutopic and control endometrium using xCell analysis based on the dataset GSE78851. Student’s t-test was used to calculate the significant changes between groups, with one asterisk (*) indicating p-value < 0.05 and “ns” standing for no significant difference between two groups. **(B)** Boxplot comparing the immune cell proportions between eutopic and control endometrium using CIBERSORT analysis based on the dataset GSE193928. Student’s t-test was used to calculate the significant changes between groups, with one asterisk (*) indicating p-value < 0.05, double asterisks (**) indicating p-value < 0.01 and “ns” standing for no significant difference between two groups.

Supplementary Figure 3 **(A–F)** Boxplot illustrating the distinct activity of metabolic pathways in the eutopic endometrium and the controls. The y-axis stands for the pathway scores calculated by METAFlux analysis. The significance changes were calculated with Student’s t-test, with a single asterisk (*) indicating p-value < 0.05 and double asterisks (**) indicating p-value < 0.01.

Supplementary Figure 4 The UMAP plots displaying the expression of selected marker genes across endometrial cell clusters. Each dot represents a single cell, and the color intensity corresponds to the expression level of the respective gene.

Supplementary Figure 5 Heatmap showing the activity of reactions involved in keratan sulfate biosynthesis across different cell type. Each row stands for one cell type, and each column stands for one reaction. The intensity of the color reflects the scaled flux score of different reactions, with red indicating higher activity and blue indicating lower activity.

Supplementary Figure 6 **(A)** Correlation between the activity of keratan sulfate degradation and the proportion of M2 calculated by Spearman correlation analysis. The y-axis represents the pathway score calculated by METAFlux, and the x-axis indicates the proportion of M2 macrophages among total macrophages. The correlation coefficient (R) and p-value are shown on the plot. **(B)** Boxplot illustrating the activity of keratan sulfate degradation across different cell types. Statistical significance was assessed using one-way ANOVA. **(C)** Heatmap showing the activity of reactions involved in keratan sulfate degradation across different cell type. Each row stands for one cell type, and each column stands for one reaction. The intensity of the color reflects the scaled flux score of different reactions, with red indicating higher activity and blue indicating lower activity. **(D)** Heatmap displaying the flux of keratan sulfate (products) across different cell type. Color represents the direction of flux, with red representing release and blue indicating uptake. The intensity of the color reflects the scaled flux score of different products.

## References

[1] Benagiano G Habiba M Brosens I . The pathophysiology of uterine adenomyosis: an update. Fertil. Steril. (2012) 98:572–9. doi: 10.1016/j.fertnstert.2012.06.044, PMID: 22819188

[2] Kho KA Chen JS Halvorson LM . Diagnosis, evaluation, and treatment of adenomyosis. JAMA. (2021) 326:177–8. doi: 10.1001/jama.2020.26436, PMID: 34255015

[3] Garcia-Solares J Donnez J Donnez O Dolmans M . Pathogenesis of uterine adenomyosis: invagination or metaplasia? Fertil. Steril. (2018) 109:371–9. doi: 10.1016/j.fertnstert.2017.12.030, PMID: 29566849

[4] Bourdon M Santulli P Jeljeli M Vannuccini S Marcellin L Doridot L . Immunological changes associated with adenomyosis: a systematic review. Hum Reprod Update. (2021) 27:108–29. doi: 10.1093/humupd/dmaa038, PMID: 33099635

[5] Capobianco A Monno A Cottone L Venneri MA Biziato D Di Puppo F . Proangiogenic tie2(+) macrophages infiltrate human and murine endometriotic lesions and dictate their growth in a mouse model of the disease. Am J Pathol. (2011) 179:2651–9. doi: 10.1016/j.ajpath.2011.07.029, PMID: 21924227 PMC3204092

[6] An M Li D Yuan M Li Q Zhang L Wang G . Interaction of macrophages and endometrial cells induces epithelial-mesenchymal transition-like processes in adenomyosis. Biol Reprod. (2017) 96:46–57. doi: 10.1095/biolreprod.116.144071, PMID: 28395325

[7] Niu W Zhang Y Liu H Liang N Xu L Li Y . Single-cell profiling uncovers the roles of endometrial fibrosis and microenvironmental changes in adenomyosis. J Inflamm Res. (2023) 16:1949–65. doi: 10.2147/JIR.S402734, PMID: 37179754 PMC10167994

[8] Jones RK Bulmer JN Searle RF . Phenotypic and functional studies of leukocytes in human endometrium and endometriosis. Hum Reprod Update. (1998) 4:702–9. doi: 10.1093/humupd/4.5.702, PMID: 10027623

[9] Tremellen KP Russell P . The distribution of immune cells and macrophages in the endometrium of women with recurrent reproductive failure. II: adenomyosis and macrophages. J Reprod Immunol. (2012) 93:58–63. doi: 10.1016/j.jri.2011.12.001, PMID: 22209314

[10] Yang J Chen M Chen H Lee T Ho H Yang Y . Decreased expression of killer cell inhibitory receptors on natural killer cells in eutopic endometrium in women with adenomyosis. Hum Reprod. (2004) 19:1974–8. doi: 10.1093/humrep/deh372, PMID: 15217996

[11] Chen Y Li H Chang Y Yuan C Tai L Lu KH . Suppression of migratory/invasive ability and induction of apoptosis in adenomyosis-derived mesenchymal stem cells by cyclooxygenase-2 inhibitors. Fertil. Steril. (2010) 94:1972–9. doi: 10.1016/j.fertnstert.2010.01.070, PMID: 20227073

[12] Sotnikova N Antsiferova I Malyshkina A . Cytokine network of eutopic and ectopic endometrium in women with adenomyosis. Am J Reprod Immunol. (2002) 47:251–5. doi: 10.1034/j.1600-0897.2002.01040.x, PMID: 12069392

[13] Wang F Li H Yang Z Du X Cui M Wen Z . Expression of interleukin-10 in patients with adenomyosis. Fertil. Steril. (2009) 91:1681–5. doi: 10.1016/j.fertnstert.2008.02.164, PMID: 18439592

[14] Tokarz J Adamski J Rizner TL . Metabolomics for diagnosis and prognosis of uterine diseases? A systematic review. J Pers. Med. (2020) 10:8–12. doi: 10.3390/jpm10040294, PMID: 33371433 PMC7767462

[15] Yang H Lau WB Lau B Xuan Y Zhou S Zhao L . A mass spectrometric insight into the origins of benign gynecological disorders. Mass Spectrom. Rev. (2017) 36:450–70. doi: 10.1002/mas.21484, PMID: 26633258

[16] Bourdon M Santulli P Kateb F Pocate-Cheriet K Batteux F Maignien C . Adenomyosis is associated with specific proton nuclear magnetic resonance ((1)h-nmr) serum metabolic profiles. Fertil. Steril. (2021) 116:243–54. doi: 10.1016/j.fertnstert.2021.02.031, PMID: 33849709

[17] Song W Zhang Z Jiang Y Cao Y Zhang B Wang Y . Integrative metabolomic profiling reveals aberrations in myometrium associated with adenomyosis: a pilot study. Reprod Biol Endocrinol. (2022) 20:49. doi: 10.1186/s12958-022-00914-5, PMID: 35264202 PMC8905769

[18] DeBerardinis RJ . Tumor microenvironment, metabolism, and immunotherapy. N Engl J Med. (2020) 382:869–71. doi: 10.1056/NEJMcibr1914890, PMID: 32101671

[19] Leone RD Zhao L Englert JM Sun I Oh M Sun I . Glutamine blockade induces divergent metabolic programs to overcome tumor immune evasion. Science. (2019) 366:1013–21. doi: 10.1126/science.aav2588, PMID: 31699883 PMC7023461

[20] Yan C Zheng L Jiang S Yang H Guo J Jiang L . Exhaustion-associated cholesterol deficiency dampens the cytotoxic arm of antitumor immunity. Cancer Cell. (2023) 41:1276–93. doi: 10.1016/j.ccell.2023.04.016, PMID: 37244259

[21] Patro R Duggal G Love MI Irizarry RA Kingsford C . Salmon provides fast and bias-aware quantification of transcript expression. Nat Methods. (2017) 14:417–9. doi: 10.1038/nmeth.4197, PMID: 28263959 PMC5600148

[22] Wu T Hu E Xu S Chen M Guo P Dai Z . Clusterprofiler 4.0: a universal enrichment tool for interpreting omics data. Innovation (Camb). (2021) 2:100141. doi: 10.1016/j.xinn.2021.100141, PMID: 34557778 PMC8454663

[23] Kanehisa M Goto S . KEGG: kyoto encyclopedia of genes and genomes. Nucleic. Acids Res. (2000) 28:27–30. doi: 10.1093/nar/28.1.27, PMID: 10592173 PMC102409

[24] Agrawal A Balci H Hanspers K Coort SL Martens M Slenter DN . Wikipathways 2024: next generation pathway database. Nucleic. Acids Res. (2024) 52:D679–89. doi: 10.1093/nar/gkad960, PMID: 37941138 PMC10767877

[25] Steen CB Liu CL Alizadeh AA Newman AM . Profiling cell type abundance and expression in bulk tissues with CIBERSORTx. Methods Mol Biol. (2020) 2117:135–57. doi: 10.1007/978-1-0716-0301-7_7, PMID: 31960376 PMC7695353

[26] Aran D Hu Z Butte AJ . xCell: digitally portraying the tissue cellular heterogeneity landscape. Genome Biol. (2017) 18:220. doi: 10.1186/s13059-017-1349-1, PMID: 29141660 PMC5688663

[27] Hanzelmann S Castelo R Guinney J . GSVA: gene set variation analysis for microarray and RNA-Seq data. BMC Bioinf. (2013) 14:7. doi: 10.1186/1471-2105-14-7, PMID: 23323831 PMC3618321

[28] Mareckova M Garcia-Alonso L Moullet M Lorenzi V Petryszak R Sancho-Serra C . An integrated single-cell reference atlas of the human endometrium. Nat Genet. (2024) 56:1925–37. doi: 10.1038/s41588-024-01873-w, PMID: 39198675 PMC11387200

[29] Huang Y Mohanty V Dede M Tsai K Daher M Li L . Characterizing cancer metabolism from bulk and single-cell rna-seq data using metaflux. Nat Commun. (2023) 14:4883. doi: 10.1038/s41467-023-40457-w, PMID: 37573313 PMC10423258

[30] Herndon CN Aghajanova L Balayan S Erikson D Barragan F Goldfien G . Global transcriptome abnormalities of the eutopic endometrium from women with adenomyosis. Reprod Sci. (2016) 23:1289–303. doi: 10.1177/1933719116650758, PMID: 27233751 PMC6344825

[31] Lai Z Yang H Ha S Chang K Mei J Zhou W . Cyclooxygenase-2 in endometriosis. Int J Biol Sci. (2019) 15:2783–97. doi: 10.7150/ijbs.35128, PMID: 31853218 PMC6909960

[32] Dennis EA Norris PC . Eicosanoid storm in infection and inflammation. Nat Rev Immunol. (2015) 15:511–23. doi: 10.1038/nri3859, PMID: 26139350 PMC4606863

[33] Guo S . Cracking the enigma of adenomyosis: an update on its pathogenesis and pathophysiology. Reproduction. (2022) 164:R101–21. doi: 10.1530/REP-22-0224, PMID: 36099328

[34] Roszer T . Understanding the mysterious m2 macrophage through activation markers and effector mechanisms. Mediat. Inflamm. (2015) 2015:816460. doi: 10.1155/2015/816460, PMID: 26089604 PMC4452191

[35] Nishikoba N Kumagai K Kanmura S Nakamura Y Ono M Eguchi H . Hgf-met signaling shifts m1 macrophages toward an m2-like phenotype through pi3k-mediated induction of arginase-1 expression. Front Immunol. (2020) 11:2135. doi: 10.3389/fimmu.2020.02135, PMID: 32983173 PMC7492554

[36] Zhang F Wang H Wang X Jiang G Liu H Zhang G . Tgf-beta induces m2-like macrophage polarization via snail-mediated suppression of a pro-inflammatory phenotype. Oncotarget. (2016) 7:52294–306. doi: 10.18632/oncotarget.10561, PMID: 27418133 PMC5239552

[37] Atkins HM Bharadwaj MS O’Brien CA Furdui CM Appt SE Caudell DL . Endometrium and endometriosis tissue mitochondrial energy metabolism in a nonhuman primate model. Reprod Biol Endocrinol. (2019) 17:70. doi: 10.1186/s12958-019-0513-8, PMID: 31445519 PMC6708555

[38] Zhang D Wang Z Luo X Guo H Qiu G Gong Y . Cysteine dioxygenase and taurine are essential for embryo implantation by involving in e(2)-eralpha and p(4)-pr signaling in mouse. J Anim. Sci Biotechnol. (2023) 14:6. doi: 10.1186/s40104-022-00804-1, PMID: 36604722 PMC9814424

[39] Zhong Q Xiao X Qiu Y Xu Z Chen C Chong B . Protein posttranslational modifications in health and diseases: functions, regulatory mechanisms, and therapeutic implications. MedComm. (2023) 4:e261. doi: 10.1002/mco2.261, PMID: 37143582 PMC10152985

[40] Graham RA Li TC Cooke ID Aplin JD . Keratan sulphate as a secretory product of human endometrium: cyclic expression in normal women. Hum Reprod. (1994) 9:926–30. doi: 10.1093/oxfordjournals.humrep.a138618, PMID: 7929743

[41] Wang B Wu L Chen J Dong L Chen C Wen Z . Metabolism pathways of arachidonic acids: mechanisms and potential therapeutic targets. Signal Transduction Targeting Ther. (2021) 6:94. doi: 10.1038/s41392-020-00443-w, PMID: 33637672 PMC7910446

[42] Stratopoulou CA Cussac S D’Argent M Donnez J Dolmans M . M2 macrophages enhance endometrial cell invasiveness by promoting collective cell migration in uterine adenomyosis. Reprod Biomed Online. (2023) 46:729–38. doi: 10.1016/j.rbmo.2023.01.001, PMID: 36792417

[43] Mantovani A Allavena P Marchesi F Garlanda C . Macrophages as tools and targets in cancer therapy. Nat Rev Drug Discov. (2022) 21:799–820. doi: 10.1038/s41573-022-00520-5, PMID: 35974096 PMC9380983

[44] Sheu KM Hoffmann A . Functional hallmarks of healthy macrophage responses: their regulatory basis and disease relevance. Annu Rev Immunol. (2022) 40:295–321. doi: 10.1146/annurev-immunol-101320-031555, PMID: 35471841 PMC10074967

[45] Maheshwari A Kelly DR Nicola T Ambalavanan N Jain SK Murphy-Ullrich J . Tgf-beta2 suppresses macrophage cytokine production and mucosal inflammatory responses in the developing intestine. Gastroenterology. (2011) 140:242–53. doi: 10.1053/j.gastro.2010.09.043, PMID: 20875417 PMC3008335

[46] Sanjeev D Dagamajalu S Shaji V George M Subbannayya Y Prasad TSK . A network map of macrophage-stimulating protein (msp) signaling. J Cell Commun Signal. (2023) 17:1113–20. doi: 10.1007/s12079-023-00755-w, PMID: 37142846 PMC10409925

[47] Li M Yang Y Xiong L Jiang P Wang J Li C . Metabolism, metabolites, and macrophages in cancer. J Hematol Oncol. (2023) 16:80. doi: 10.1186/s13045-023-01478-6, PMID: 37491279 PMC10367370

[48] Subramanian A Tamayo P Mootha VK Mukherjee S Ebert BL Gillette MA . Gene set enrichment analysis: a knowledge-based approach for interpreting genome-wide expression profiles. Proc Natl Acad Sci U. S. A. (2005) 102:15545–50. doi: 10.1073/pnas.0506580102, PMID: 16199517 PMC1239896

[49] Sarsenova M Lawarde A Pathare ADS Saare M Modhukur V Soplepmann P . Endometriotic lesions exhibit distinct metabolic signature compared to paired eutopic endometrium at the single-cell level. Commun Biol. (2024) 7:1026. doi: 10.1038/s42003-024-06713-5, PMID: 39169201 PMC11339455

[50] Zhang H Zhang C Zhang S . Single-cell RNA transcriptome of the human endometrium reveals epithelial characterizations associated with recurrent implantation failure. Adv Biol. (2024) 8:e2300110. doi: 10.1002/adbi.202300110, PMID: 37690851

[51] Hoadley ME Seif MW Aplin JD . Menstrual-cycle-dependent expression of keratan sulphate in human endometrium. Biochem J. (1990) 266:757–63. doi: 10.1042/bj2660757, PMID: 1691631 PMC1131204

[52] Wolanska M Sobolewski K Drozdzewicz M Bankowski E . Extracellular matrix components in uterine leiomyoma and their alteration during the tumour growth. Mol Cell Biochem. (1998) 189:145–52. doi: 10.1023/a:1006914301565, PMID: 9879665

[53] Cui Y Li J Zhang P Yin D Wang Z Dai J . B4galt1 promotes immune escape by regulating the expression of pd-l1 at multiple levels in lung adenocarcinoma. J Exp Clin Cancer Res. (2023) 42:146. doi: 10.1186/s13046-023-02711-3, PMID: 37303063 PMC10259029

[54] Joung J Kirchgatterer PC Singh A Cho JH Nety SP Larson RC . CRISPR activation screen identifies BCL-2 proteins and B3GNT2 as drivers of cancer resistance to t cell-mediated cytotoxicity. Nat Commun. (2022) 13:1606. doi: 10.1038/s41467-022-29205-8, PMID: 35338135 PMC8956604

[55] Bull C Nason R Sun L Van Coillie J Madriz SD Moons SJ . Probing the binding specificities of human siglecs by cell-based glycan arrays. Proc Natl Acad Sci U. S. A. (2021) 118:6–9. doi: 10.1073/pnas.2026102118, PMID: 33893239 PMC8092401

[56] Zheng T Zhao C Zhao B Liu H Wang S Wang L . Impairment of the autophagy-lysosomal pathway and activation of pyroptosis in macular corneal dystrophy. Cell Death Discov. (2020) 6:85. doi: 10.1038/s41420-020-00320-z, PMID: 32983576 PMC7487068

