# Correction: Metabolic reprogramming and M2 macrophage depletion define the microenvironment of adenomyosis

**DOI:** 10.3389/fendo.2026.1809151

**Published:** 2026-02-18

**Authors:** Frontiers Production Office

**Keywords:** adenomyosis, endometrium, immune infiltration, macrophage, metabolic flux, Keratan sulfate

Affiliation “Instituto de Investigación Sanitaria La Fe/Fundación IVI, Spain” for Editor Hortensia Ferrero was erroneously given as “Valencian Institute for Agricultural Research (IVIA), Spain”.

The original version of this article has been updated.

