# Supplementary figures and images for: Metabolic reprogramming and M2 macrophage depletion define the microenvironment of adenomyosis
Source: Front Endocrinol (Lausanne). 2025 Nov 20;16:1602814. doi: 10.3389/fendo.2025.1602814 (PMC12675163; doi:10.3389/fendo.2025.1602814)

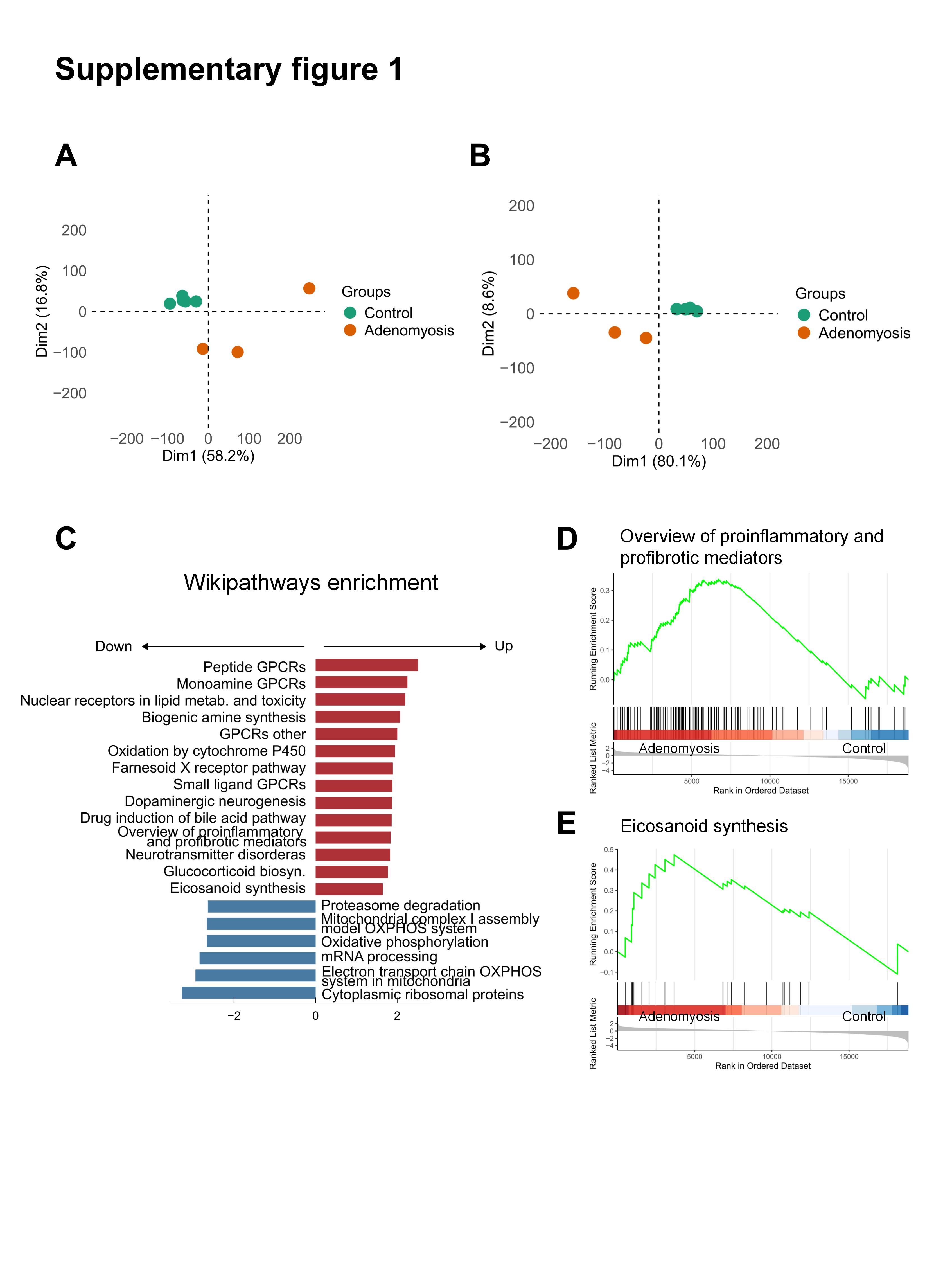

Supplement: Supplementary Figure 1 — (A) Principal component analysis of the microarray dataset GSE78851 based on all detected genes. (B) Principal component analysis of the microarrary dataset GSE78851 based on the identified DEGs. (C) The bidirectional barplot showing GSEA results based on the Wikipathways database. The x-axis represents the normalized enrichment scores (NES). The positive value indicates that the pathways are enriched in the adenomyosis group, while the negative value indicates that the pathways are enriched in the control group. (D, E) GSEA plots displaying the enrichment of overview of proinflammatory and profibrotic mediators (D), eicosanoid synthesis (E). The enrichment score indicates the pathway activity in the adenomyosis versus control. [file Image1.jpeg]

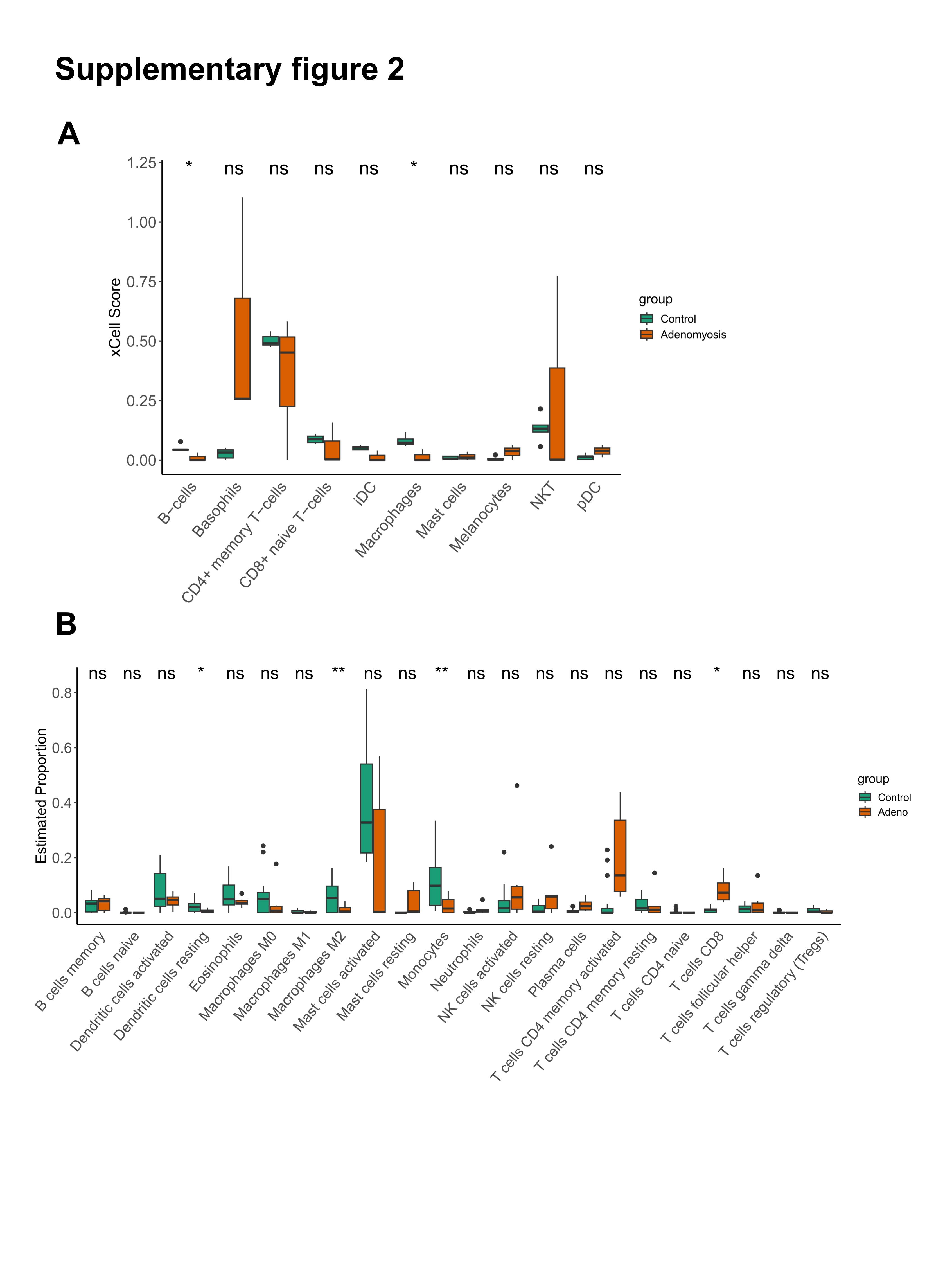

Supplement: Supplementary Figure 2 — (A) Boxplot comparing the immune cell infiltration score between eutopic and control endometrium using xCell analysis based on the dataset GSE78851. Student’s t-test was used to calculate the significant changes between groups, with one asterisk (*) indicating p-value < 0.05 and “ns” standing for no significant difference between two groups. (B) Boxplot comparing the immune cell proportions between eutopic and control endometrium using CIBERSORT analysis based on the dataset GSE193928. Student’s t-test was used to calculate the significant changes between groups, with one asterisk (*) indicating p-value < 0.05, double asterisks (**) indicating p-value < 0.01 and “ns” standing for no significant difference between two groups. [file Image2.jpeg]

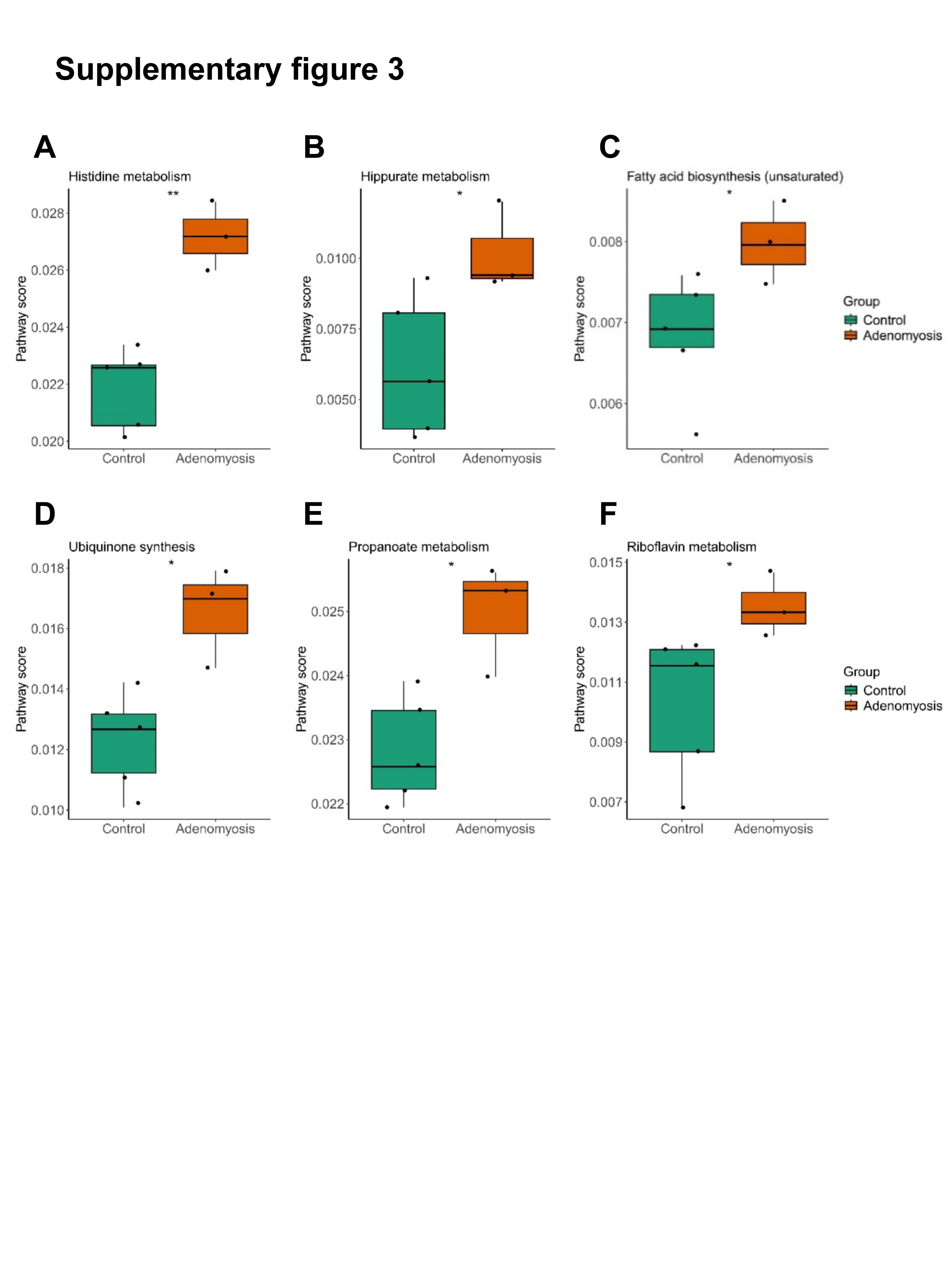

Supplement: Supplementary Figure 3 — (A–F) Boxplot illustrating the distinct activity of metabolic pathways in the eutopic endometrium and the controls. The y-axis stands for the pathway scores calculated by METAFlux analysis. The significance changes were calculated with Student’s t-test, with a single asterisk (*) indicating p-value < 0.05 and double asterisks (**) indicating p-value < 0.01. [file Image3.jpeg]

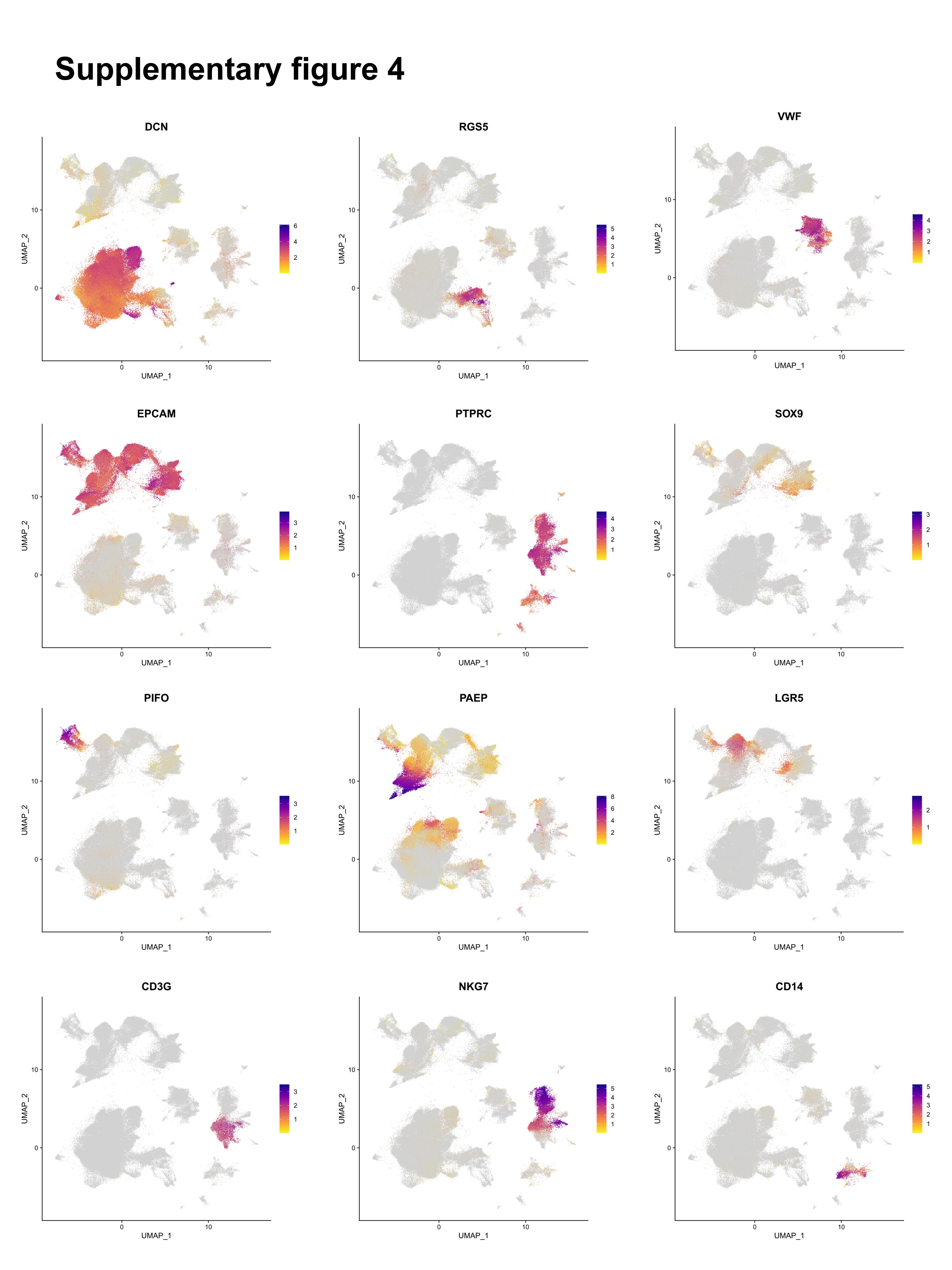

Supplement: Supplementary Figure 4 — The UMAP plots displaying the expression of selected marker genes across endometrial cell clusters. Each dot represents a single cell, and the color intensity corresponds to the expression level of the respective gene. [file Image4.jpeg]

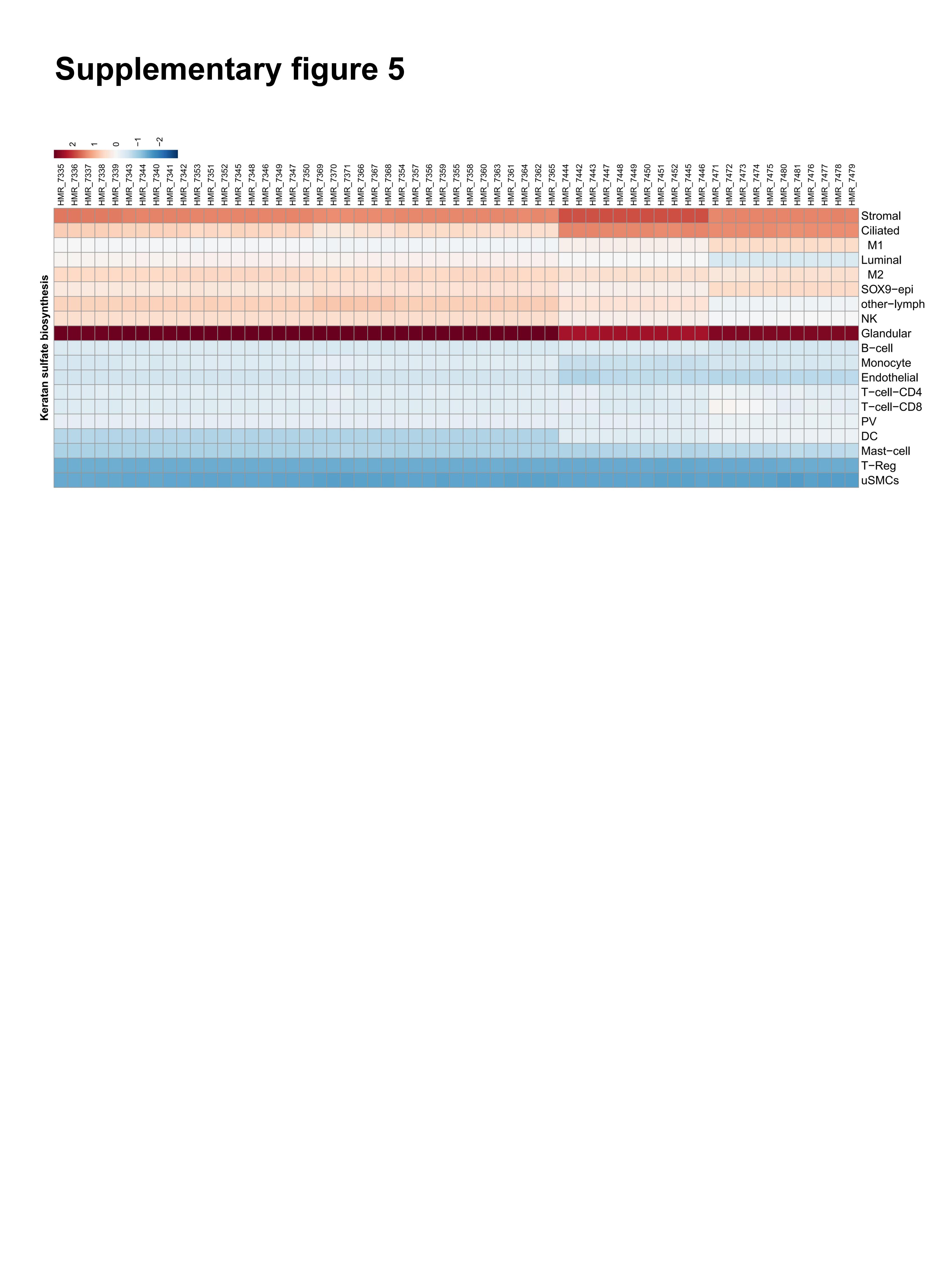

Supplement: Supplementary Figure 5 — Heatmap showing the activity of reactions involved in keratan sulfate biosynthesis across different cell type. Each row stands for one cell type, and each column stands for one reaction. The intensity of the color reflects the scaled flux score of different reactions, with red indicating higher activity and blue indicating lower activity. [file Image5.jpeg]

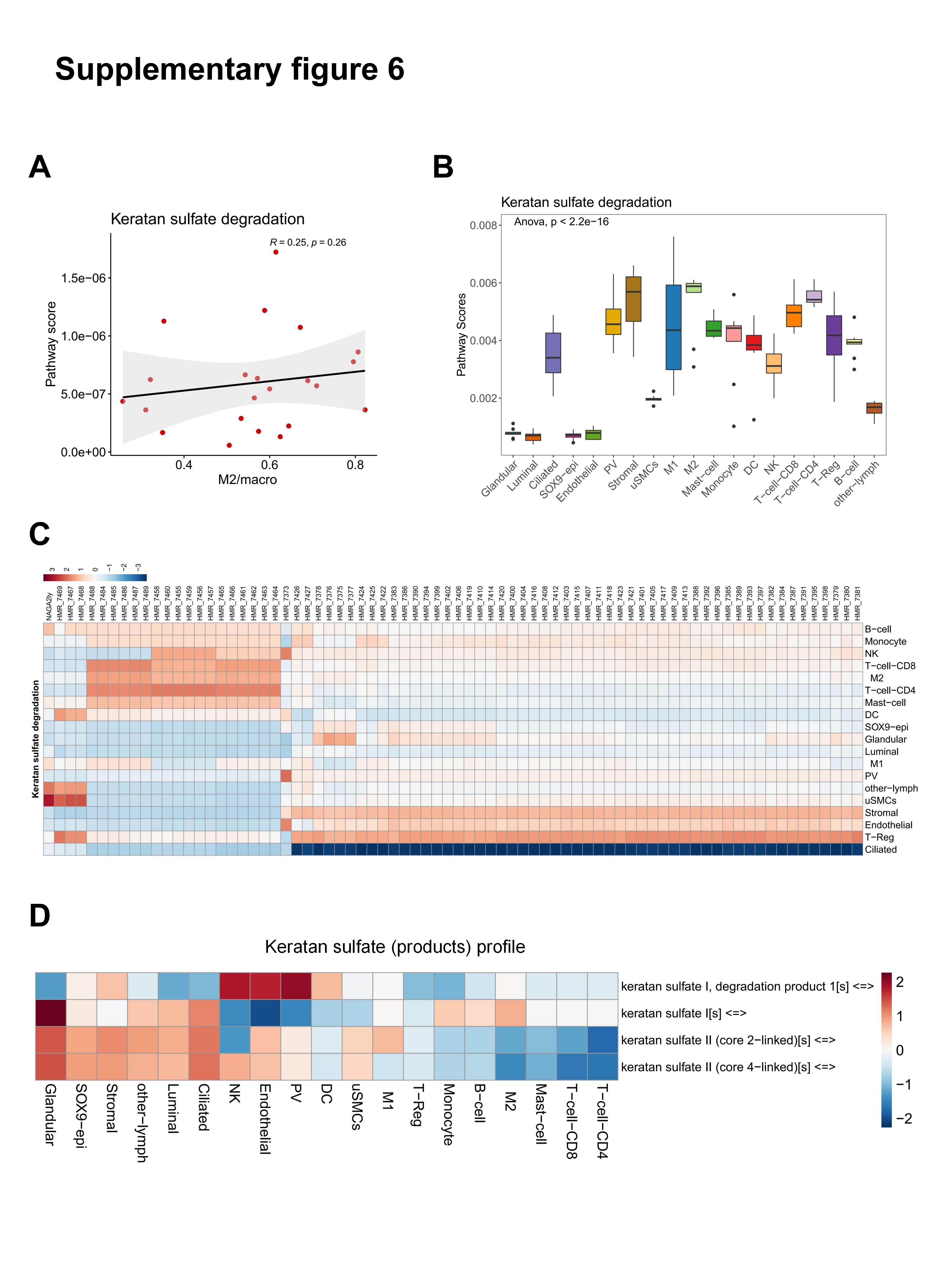

Supplement: Supplementary Figure 6 — (A) Correlation between the activity of keratan sulfate degradation and the proportion of M2 calculated by Spearman correlation analysis. The y-axis represents the pathway score calculated by METAFlux, and the x-axis indicates the proportion of M2 macrophages among total macrophages. The correlation coefficient (R) and p-value are shown on the plot. (B) Boxplot illustrating the activity of keratan sulfate degradation across different cell types. Statistical significance was assessed using one-way ANOVA. (C) Heatmap showing the activity of reactions involved in keratan sulfate degradation across different cell type. Each row stands for one cell type, and each column stands for one reaction. The intensity of the color reflects the scaled flux score of different reactions, with red indicating higher activity and blue indicating lower activity. (D) Heatmap displaying the flux of keratan sulfate (products) across different cell type. Color represents the direction of flux, with red representing release and blue indicating uptake. The intensity of the color reflects the scaled flux score of different products. [file Image6.jpeg]
